# Effect of educational program for patients with type 2 diabetes: a systematic review and meta-analysis of randomized controlled trials

**DOI:** 10.3389/fmed.2026.1860805

**Published:** 2026-08-13

**Authors:** Xiao Cheng, Yan Shi

**Affiliations:** Department of Nursing, Shanghai Baoshan District Zhangmiao Street Sitang Community Health Service Center, Shanghai, China

**Keywords:** glycated hemoglobin, meta-analysis, structured education programs, systematic review, type diabetes

## Abstract

**Objectives:**

Type 2 diabetes (T2D) remains a major global health challenge, with effective disease management largely dependent on patient self-care. Although structured patient education is a cornerstone intervention, a comprehensive and updated synthesis of its effects on glycemic and cardiometabolic outcomes is still required. This systematic review and meta-analysis aimed to evaluate the efficacy of structured education programs vs. usual care on clinical outcomes in adults with T2D.

**Methods:**

We systematically searched PubMed, Embase, the Cochrane Central Register of Controlled Trials (CENTRAL), and Web of Science from their inception to December 2025. The primary outcome was the change in glycated hemoglobin (HbA1c). Two reviewers independently assessed the risk of bias in included studies using the revised Cochrane Risk of Bias 2 (ROB 2) tool. Pooled weighted mean differences (WMDs) with 95% confidence intervals (CIs) were calculated using random-effects models. Subgroup, sensitivity, and publication bias analyses were also conducted.

**Results:**

Of 16,891 records screened, 49 randomized controlled trials involving 9,587 participants (sample sizes ranging from 53 to 1,489 and follow-up durations from 3 months to 7.7 years) were included. Compared with usual care, structured education programs significantly reduced HbA1c (WMD: −0.55%; 95% CI: −0.70 to −0.39), fasting blood glucose (WMD: −1.35 mmol/L; 95% CI: −1.86 to −0.84; *P* < 0.001), 2–h postprandial glucose (WMD: −1.17 mmol/L; 95% CI: −1.99 to −0.34; *P* = 0.005), body mass index (WMD: −0.41 kg/m^2^; 95% CI: −0.71 to −0.11), systolic blood pressure (WMD: −3.76 mmHg; 95% CI: −6.08 to −1.43), diastolic blood pressure (WMD: −2.21 mmHg; 95% CI: −3.90 to −0.52), low–density lipoprotein cholesterol (WMD: −10.61 mg/dL; 95% CI: −16.25 to −4.98), total cholesterol (WMD: −12.07 mg/dL; 95% CI: −16.97 to −7.17), and triglycerides (WMD: −12.69 mg/dL; 95% CI: −18.24 to −7.14). No significant effect was observed on high-density lipoprotein cholesterol.

**Conclusion:**

Structured education programs are effective in improving glycemic control and multiple cardiometabolic risk factors in patients with T2D. These findings strongly support the integration of standardized patient education as a core component of comprehensive T2D management in clinical practice and guideline recommendations.

**Systematic review registration:**

The systematic review was registered in INPLASY perform, with the number of INPLASY202620043. The website was https://inplasy.com/inplasy-2026-2-0043/

## Introduction

Type 2 diabetes (T2D) represents one of the most critical global public health challenges of the 21st century, with its prevalence having risen dramatically in recent decades (1). According to the International Diabetes Federation, an estimated 589 million adults worldwide live with diabetes—a number projected to increase to 853 million by 2050 (2). More than 90% of these cases are T2D, imposing substantial economic and social burdens on healthcare systems globally (3, 4).

T2D is a complex chronic metabolic disorder whose effective management relies not only on timely pharmacological treatment but also, to a large extent, on sustained, evidence-based self-management by patients. Essential self-management behaviors include regular blood glucose monitoring, dietary adherence, engagement in physical activity, appropriate medication use, and consistent foot care (5, 6). In clinical practice, however, many individuals with T2D fail to achieve optimal metabolic control. This directly elevates the risk of developing microvascular (e.g., retinopathy, nephropathy, neuropathy) and macrovascular (e.g., cardiovascular disease) complications, significantly impairing quality of life and reducing life expectancy (7, 8).

A key contributor to this gap is insufficient patient knowledge and skills related to self-management. When confronted with complex disease information and long-term health decisions, patients often experience confusion, helplessness, or inertia, leading to suboptimal treatment adherence (9). Empowering patients and enhancing their self-management capacity are therefore crucial strategies for improving clinical outcomes in T2D.

Structured patient education programs are widely regarded as foundational interventions for achieving this goal (10). Typically designed and delivered by multidisciplinary teams, such programs employ systematic curricula, interactive workshops, or personalized coaching to provide disease-related knowledge, skill training, and psychosocial support. They aim to foster healthy behaviors and strengthen patients' confidence and self-efficacy in managing their condition (11).

Although numerous observational studies and randomized controlled trials (RCTs) have examined the benefits of education programs for people with T2D, findings remain inconsistent. Previous systematic reviews have provided valuable insights but have several important limitations that justify an updated synthesis. First, many earlier reviews focused narrowly on glycemic control (HbA1c) as the primary outcome, with limited or inconsistent reporting of other clinically important cardiometabolic outcomes such as blood pressure, lipid profiles, and weight management (12). Second, the rapid proliferation of digital health interventions—including mobile apps, telemedicine, and web-based platforms—has introduced new delivery modalities that were not adequately represented in earlier reviews (13). Third, previous reviews often did not systematically examine intervention characteristics that may moderate treatment effects, limiting their utility for guiding intervention design (14). Fourth, the continuous publication of new high-quality RCTs—provides an opportunity to update the evidence base with greater precision and to explore sources of heterogeneity more thoroughly. Therefore, this updated systematic review and meta-analysis aims to provide a comprehensive and methodologically rigorous synthesis of the effects of structured education programs on multiple clinical outcomes in individuals with T2D, with particular attention to identifying factors that moderate intervention effectiveness.

## Materials and methods

### Search strategy and selection criteria

This systematic review and meta-analysis was conducted in accordance with the Preferred Reporting Items for Systematic Reviews and Meta-Analyses (PRISMA 2020) guidelines (15). The study protocol was registered on INPLASY (registration number: INPLASY202620043).

A comprehensive systematic search was performed in PubMed, Embase, the Cochrane Central Register of Controlled Trials (CENTRAL), and Web of Science from their inception to December 31, 2025. The search strategy combined subject headings (e.g., MeSH, Emtree) and free-text terms centered on three key concepts: “type 2 diabetes,” “patient education,” and “randomized controlled trial.” The detailed search strategies for each database are provided in Supplementary File 1. In addition to the database searches, we manually screened the reference lists of all included studies and relevant previous systematic reviews to identify any additional potentially eligible studies not captured by the electronic searches. No language restrictions were applied.

Literature search and study selection were carried out independently by two investigators. Disagreements were resolved through discussion or, when necessary, by consultation with a third investigator. Studies were included if they met the following criteria: (1) study design: randomized controlled trials (RCTs), including cluster RCTs; (2) participants: adults (age ≥18 years) clinically diagnosed with type 2 diabetes, with no restrictions on disease duration, baseline glycated hemoglobin (HbA1c), or comorbidities; (3) intervention: the experimental group received any form of structured, systematic diabetes education program covering one or more aspects of disease knowledge and self-management skills (e.g., diet, physical activity, medication adherence, glucose monitoring), delivered via face-to-face sessions, group workshops, remote education, or a blended format; (4) comparator: the control group received usual care, routine clinic follow-up, a waitlist, or minimal standard health education; and (5) outcomes: at least one of the following primary or secondary outcomes was reported: change in HbA1c, fasting blood glucose (FBG) and 2-h postprandial glucose (2hPG), body mass index (BMI), systolic blood pressure (SBP), diastolic blood pressure (DBP), or lipid profile [total cholesterol (TC), low-density lipoprotein cholesterol (LDL-C), high-density lipoprotein cholesterol (HDL-C), triglycerides (TG)]. In cases where multiple publications from the same randomized controlled trial were identified (i.e., affiliated trials reporting on different outcomes, follow-up time points, or subgroups), we included only the primary report—defined as the publication reporting the primary outcome or the most comprehensive description of the trial—and excluded all secondary or satellite publications to avoid double-counting participants and ensure independence of effect estimates.

Studies were excluded if they: were non-randomized; exclusively enrolled patients with type 1 diabetes, gestational diabetes, or other specific types of diabetes (studies with mixed populations were included only if outcomes for the type 2 diabetes subgroup were reported separately, or if the proportion of non-type 2 diabetes participants was negligible and unlikely to affect the overall results); involved interventions limited solely to pharmacological treatment, nutrition, or exercise prescription without systematic education or skill-training components; or did not report relevant outcome data or provided data that could not be extracted.

### Data collection and quality assessment

For included studies, two investigators independently extracted data using a pre-designed standardized form in Microsoft Excel (Microsoft Corporation, Redmond, WA, USA). Disagreements were resolved through discussion or consultation with a third investigator. Extracted information included first author, publication year, country, sample size (intervention/control groups), participant characteristics (age, sex, BMI, baseline HbA1c), detailed descriptions of the intervention and comparator, intervention characteristics (interventionists, theoretical framework, additional techniques such as motivational interviewing, frequency, session duration, total sessions, delivery mode, and education content), follow-up duration, and outcome data. Where data were missing or unclear, corresponding authors were contacted via email to request the necessary information. The risk of bias for each included RCT was independently assessed by two reviewers using the revised Cochrane Risk of Bias 2 (RoB 2) tool (16), which evaluates five domains: (1) bias arising from the randomization process, (2) bias due to deviations from intended interventions, (3) bias due to missing outcome data, (4) bias in measurement of the outcome, and (5) bias in selection of the reported result. Each domain was rated as “low risk,” “some concerns,” or “high risk,” and an overall risk of bias judgment was assigned based on the domain-level assessments. The results of the risk of bias assessment are presented visually using traffic light plots and weighted summary bar plots. Disagreements were resolved through discussion or arbitration by a third reviewer.

### Statistical analysis

For continuous outcomes, the weighted mean difference (WMD) was used as the effect measure. A random-effects model was employed to calculate pooled effect estimates and 95% confidence intervals (CIs), accounting for anticipated heterogeneity across studies in intervention characteristics, populations, and settings (17, 18). Heterogeneity was assessed using the *I*^2^ statistic and the Q-test *p*-value. *I*^2^ ≤ 25% was considered low heterogeneity, 25% < *I*^2^ ≤ 50% moderate heterogeneity, and *I*^2^ > 50% high heterogeneity. A Q-test *p*-value < 0.10 indicated significant statistical heterogeneity (19, 20). To evaluate the robustness of the findings, a leave-one-out sensitivity analysis was performed by sequentially excluding each study and observing changes in the pooled effect size (21). To explore potential sources of heterogeneity, we conducted meta-regression analyses for the primary outcome (HbA1c) to examine the association between continuous and categorical study-level characteristics and the magnitude of the intervention effect. Candidate covariates included sample size, mean age, proportion of males, mean BMI, baseline HbA1c, follow-up duration, country region, delivery mode, session type, theory-based intervention, and multidisciplinary involvement. Then the following subgroup analyses were planned for the primary outcome (HbA1c): country (Eastern vs. Western), sample size ( ≤ 100 vs. >100), mean age ( ≤ 60.0 vs. >60.0 years), proportion of males ( ≤ 60% vs. >60%), BMI ( ≤ 30 vs. >30 kg/m^2^), baseline glycemic control (HbA1c < 8.0% vs. ≥ 8.0%), delivery format (face-to-face vs. remote vs. blend), and session type (group-based vs. Individual-based), theory-based intervention (yes vs. no), multidisciplinary involvement (yes vs. no), and intervention duration ( ≤ 6 months vs. >6 months). Countries were classified as “Eastern” if located in East Asia, Southeast Asia, South Asia, or the Middle East (including China, Japan, South Korea, Malaysia, Pakistan, Indonesia, Iran, Turkey, Israel, Saudi Arabia, and the United Arab Emirates), and as “Western” if located in Europe, North America, or Australia (including the Netherlands, United Kingdom, Austria, Spain, Italy, Germany, Slovenia, Sweden, United States, Canada, and Australia). This classification was chosen based on geographical location and cultural differences in healthcare systems, patient education practices, and lifestyle patterns that may influence the effectiveness of educational interventions. Differences between subgroups were assessed using an interaction test (22). Publication bias was assessed using funnel plots, supplemented by Egger's and Begg's statistical tests (23, 24). All statistical tests were two-sided, and *p* < 0.05 was considered statistically significant. Analyses were performed using STATA version 15.0 (Stata Corporation, College Station, TX, USA).

## Results

### Literature search and study selection

**Figure 1:**
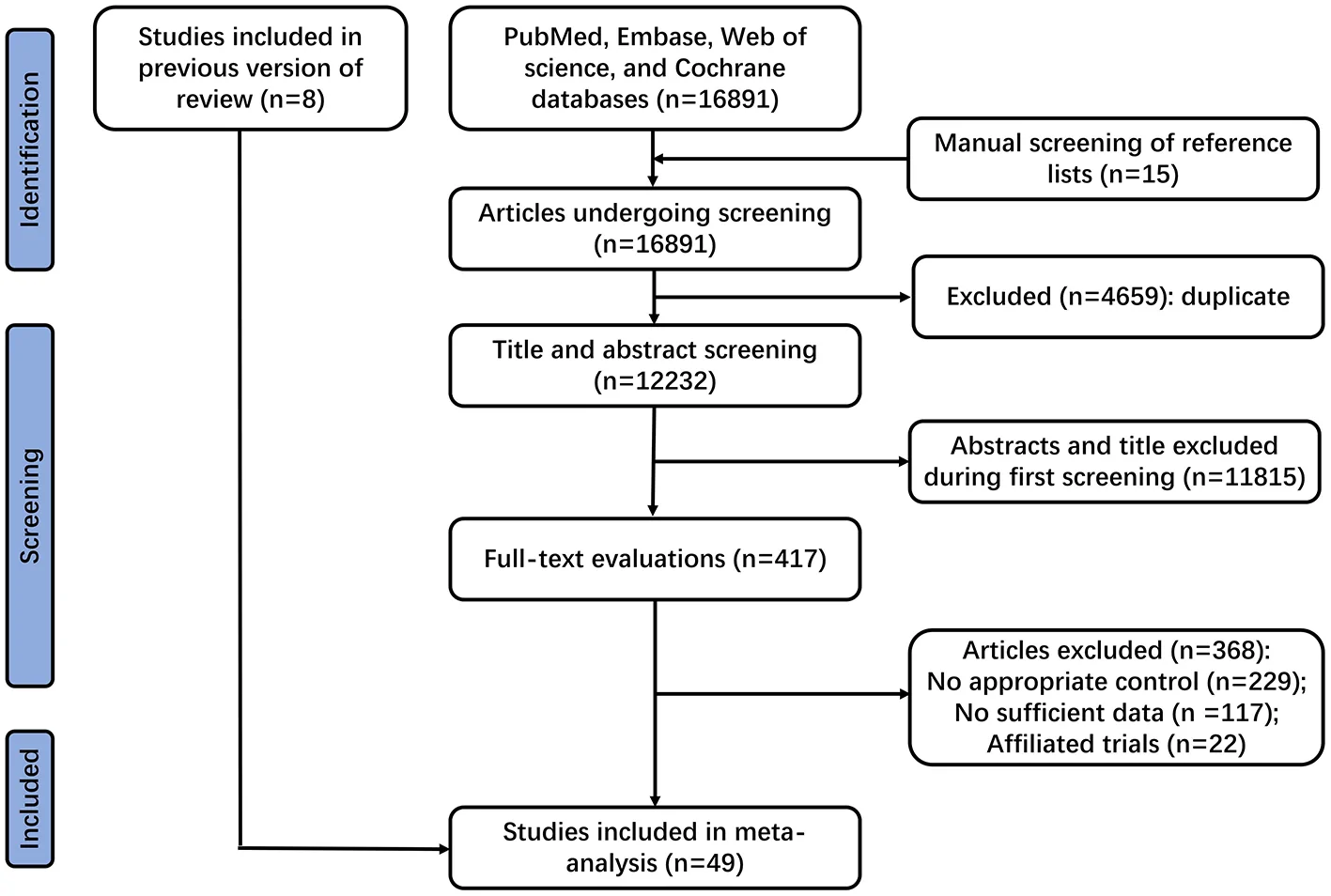
PRISMA 2020 flow diagram for updated systematic reviews that included searches of databases, registers, and other sources. The diagram shows the number of records identified, screened, assessed for eligibility, and included in the review, with reasons for exclusion at the full–text stage.

A total of 16,891 records were initially retrieved through systematic database searches. After duplicate removal, 12,232 records remained for title and abstract screening. Of these, 11,815 were excluded as clearly irrelevant. Subsequently, 417 full-text articles were assessed for eligibility. Ultimately, 49 RCTs fully met the pre-defined inclusion criteria and were included in this systematic review and meta-analysis (25–73). The complete study selection process is detailed in Figure 1.

### Trial characteristics

The 49 included RCTs involved a total of 9,587 patients with T2D and were published between 2004 and 2025 across 18 countries/regions. The distribution was as follows: Europe (*n* = 14), China (*n* = 13), Middle Eastern countries (*n* = 9), other Asian countries such as Japan and South Korea (*n* = 9), and the United States, Canada, and Australia (*n* = 4). Although no language restrictions were applied in the search strategy, all studies that met the inclusion criteria and were ultimately included in this review were published in English. Sample sizes ranged from 53 to 1,489 participants. The mean age of participants ranged from 50.5 to 73.5 years, and baseline mean HbA1c levels ranged from 7.1% to 10.9%. Follow-up duration varied from 3 months to 7.7 years. Education programs were delivered in diverse formats, including face-to-face group sessions, one-on-one education, and remote education via telephone or internet platforms. Baseline characteristics are summarized in Table 1. Detailed intervention characteristics, including interventionists, theoretical frameworks, additional techniques, delivery modes, frequency, duration, and education content, are presented in Supplementary File 2.

**Table 1:** The baseline characteristics of eligible trials and involved patients.

Trial	Country	Sample size (I/C)	Age (years)	Male (%)	BMI (kg/m^2^)	A1C (%)	Control	Follow–up
Goudswaard (25)	Netherlands	54 (25/29)	60.5	48.0	30.0	8.5	Standard consultation visits	1.5 years
Rachmani (26)	Israel	141 (71/70)	40.0–70.0	NA	NA	NA	Standard consultation visits	7.7 years
Ko (27)	Korea	437 (219/218)	53.7	43.9	25.4	9.3	Advised to undertake medical nutrition therapy	4.0 years
Shibayama (28)	Japan	134 (67/67)	61.5	65.2	25.5	7.3	Standard consultation visits	1.0 year
Cade (29)	UK	239 (112/127)	65.8	58.6	30.8	7.4	Standard consultation visits	1.0 year
Schillinger (30)	US	226 (112/114)	55.8	43.4	31.5	9.5	Standard consultation visits	1.0 year
Sonnichsen (31)	Austria	1,489 (649/840)	65.5	52.2	30.0	7.4	Standard consultation visits	1.1 year
Williams (32)	Australia	120 (60/60)	57.4	62.5	33.6	9.6	Standard consultation visits	6 months
Jarab (33)	Jordan	171 (85/86)	64.4	56.7	32.6	8.4	Standard consultation visits	6 months
Chow (34)	Saudi Arabia	150 (75/75)	60.3	36.7	NA	9.1	Standard consultation visits	3 months
Chao (35)	China	100 (50/50)	69.9	49.0	24.1	NA	Standard consultation visits	1.5 year
AI–Haj Mohd (36)	United Arab Emirates	446 (223/223)	61.5	48.5	NA	8.5	Standard consultation visits	6 months
Riddell (37)	Australia	240 (120/120)	60.9	50.8	31.8	7.3	Standard consultation visits	1.0 year
Chen (38)	China	100 (50/50)	72.4	50.0	25.8	9.1	Standard consultation visits	6 months
Ramadas (39)	Malaysia	128 (66/62)	50.5	60.2	NA	9.0	Standard consultation visits	1.0 year
Mohammadi (40)	Iran	200 (100/100)	51.3	NA	NA	8.1	Standard consultation visits	0.7 year
Tourkmani (41)	Saudi Arabia	263 (195/68)	57.1	35.0	NA	10.9	Standard consultation visits	10 months
Jiang (42)	China	265 (133/132)	56.9	44.9	24.8	8.7	Standard consultation visits	6 months
Trento (43)	Italy	50 (25/25)	62.4	64.0	28.9	7.5	Standard consultation visits	4.0 years
Guner (44)	Turkey	101 (50/51)	56.7	47.5	32.2	7.4	Standard consultation visits	6 months
De la Fuente Coria (45)	Spain	236 (97/139)	65.1	54.2	30.8	7.5	Standard consultation visits	1.0 year
Tanaka (46)	Japan	76 (38/38)	61.0	78.9	25.2	7.9	Standard consultation visits	6 months
Sanaeinasab (47)	Iran	80 (40/40)	50.7	41.0	29.2	8.9	Standard consultation visits	3 months
Pai (48)	China	108 (53/50)	66.2	47.2	NA	NA	Routine shared care	6 months
Omura (49)	Japan	126 (64/62)	58.1	73.8	26.7	8.1	Dietary advice using generalized pamphlets	6 months
Agrali (50)	Turkey	120 (60/60)	56.4	41.7	NA	7.4	Standard consultation visits	6 months
Feng (51)	China	225 (113/112)	65.6	48.4	NA	7.9	Standard consultation visits	1.0 year
Renjbar (52)	Iran	120 (60/60)	54.4	30.0	NA	7.6	Standard consultation visits	6 months
Zainudin (53)	Malaysia	180 (90/90)	56.1	46.7	29.6	10.6	Standard consultation visits	6 months
Terkes (54)	Turkey	89 (44/45)	52.2	46.1	32.4	8.3	Standard consultation visits	3 months
Asmat (55)	Pakistan	612 (302/310)	53.6	51.1	NA	8.7	Standard consultation visits	3 months
Lau (56)	Canada	105 (52/53)	57.3	50.5	32.4	8.0	Standard consultation visits	3 months
Kim (57)	Korea	106 (53/53)	58.3	49.1	26.9	8.4	Standard consultation visits	6 months
Zhang (58)	China	147 (72/75)	52.0	54.4	27.8	8.1	Standard consultation visits	3 months
Hou (59)	China	105 (53/52)	66.9	54.3	24.7	NA	Standard consultation visits	6 months
Pourmohammad (60)	Iran	60 (30/30)	54.1	26.7	NA	7.8	Standard consultation visits	3 months
Zhao (61)	China	80 (40/40)	53.7	57.5	23.3	NA	Standard consultation visits	6 months
Ye (62)	China	155 (78/77)	57.6	55.5	24.4	8.5	Standard consultation visits	6 months
Mateos (63)	Spain	85 (44/41)	53.6	56.5	37.0	9.7	Standard consultation visits	6 months
Bretschneider (64)	Germany	149 (73/76)	60.6	61.0	32.2	8.3	Standard consultation visits	6 months
Mihevc (65)	Slovenia	117 (55/62)	71.3	60.7	30.4	7.2	Standard consultation visits	1.0 year
Zhang (66)	China	198 (99/99)	73.5	49.0	NA	7.1	Standard consultation visits	6 months
Epriliawati (67)	Indonesia	53 (26/27)	52.5	30.2	28.0	8.7	Standard consultation visits	6 months
Li (68)	China	76 (38/38)	67.8	47.4	24.7	NA	Standard consultation visits	6 months
Terkes (69)	Turkey	100 (50/50)	51.3	59.0	30.4	NA	Standard consultation visits	3 months
Sjoblom (70)	Sweden	129 (67/62)	63.0	61.2	29.7	NA	Standard consultation visits	3 months
Li (71)	China	178 (90/88)	61.8	52.2	NA	8.4	Standard consultation visits	1.5 years
Isleyen (72)	Turkey	60 (30/30)	53.1	35.0	31.3	8.9	Standard consultation visits	3 months
Lo (73)	China	602 (300/302)	59.5	60.6	26.4	7.9	Standard consultation visits	3 months

### Risk of bias assessment

The risk of bias of the included studies was assessed using the revised Cochrane RoB 2 tool, with results presented visually in Supplementary File 3. Overall, 10 studies (20.4%) were rated as having low risk of bias, while 39 studies (79.6%) had some concerns. No studies were rated as high risk of bias. The primary reasons for “some concerns” ratings were: (1) inability to blind participants and personnel to educational interventions, and (2) insufficient reporting of allocation concealment methods. Regarding the randomization process domain, 32 studies (65.3%) were rated as “low risk,” while 17 studies (34.7%) had “some concerns” primarily due to insufficient description of allocation concealment or sequence generation. No studies were rated as “high risk” for this domain. For deviations from intended interventions and missing outcome data, all studies were rated as “low risk.” For outcome measurement, given the nature of educational interventions, blinding of participants was not feasible; however, 46 studies (93.9%) reported blinding of outcome assessors and were rated as “low risk,” while 3 studies (6.1%) had “some concerns” for this domain. For selection of the reported result, all studies were rated as “low risk” with pre-specified analysis plans.

### HbA1c

**Figure 2:**
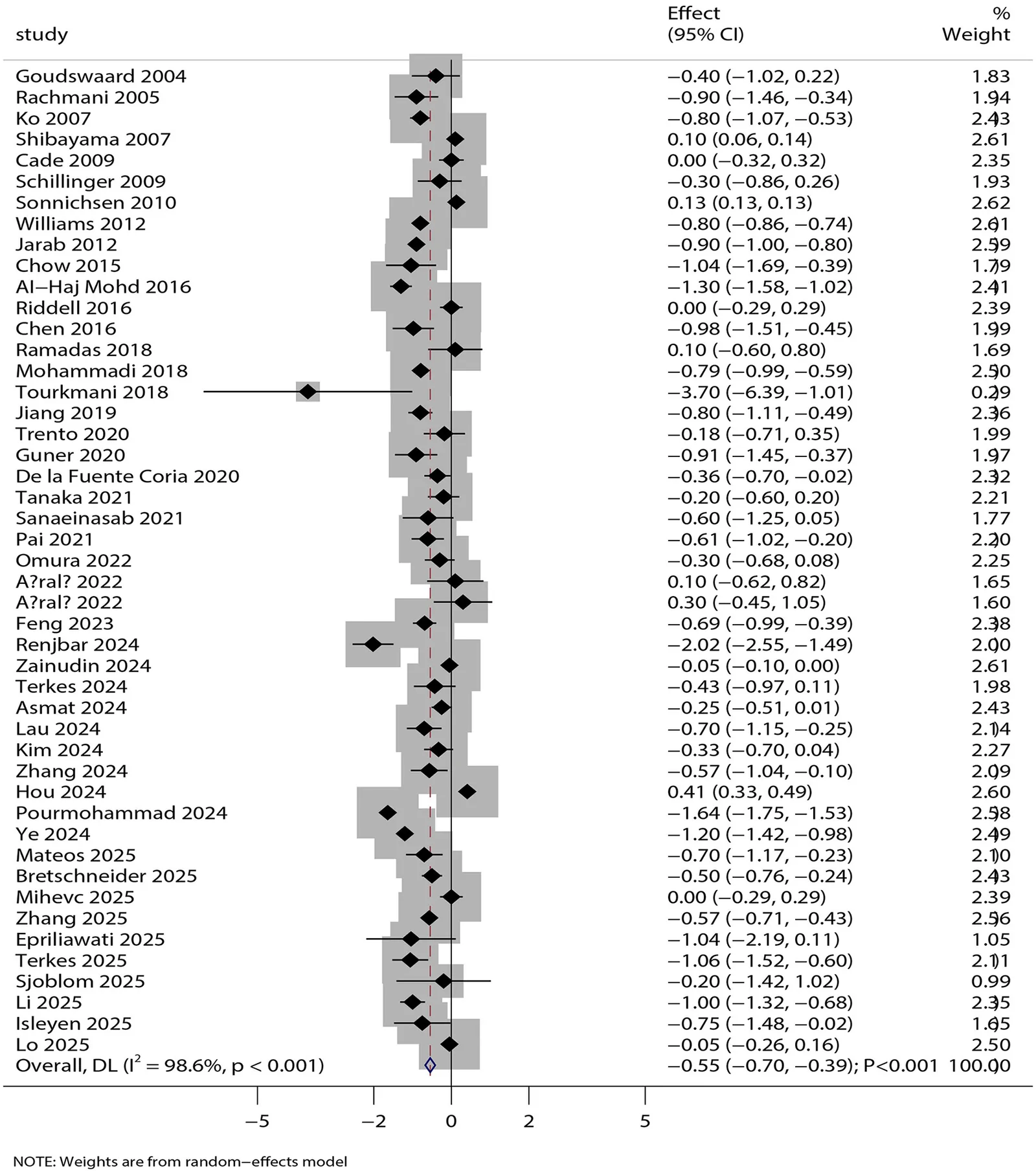
Forest plot showing the effect of structured diabetes education programs on glycated hemoglobin (HbA1c).

A total of 46 studies reported post-intervention HbA1c outcomes. Compared with the control group, patients receiving education programs showed a significant reduction in HbA1c levels (WMD: −0.55%; 95% CI: −0.70 to −0.39; *P* < 0.001; Figure 2). Considerable heterogeneity was observed among the studies (*I*^2^ = 98.6%, *p* < 0.001). Sensitivity analysis indicated that the pooled effect size remained statistically significant after removing any single study, suggesting robust findings (Supplementary File 4). To investigate potential sources of the substantial heterogeneity observed in HbA1c outcomes, we conducted meta-regression analyses. The results are presented in Supplementary File 5. We noted proportion of males (*p* = 0.001) and multidisciplinary involvement (*p* = 0.035) were significantly associated with the changes of HbA1c. Subgroup analysis demonstrated a significant reduction in HbA1c across most subgroups; however, in the subgroup with a male proportion exceeding 60%, no significant difference was observed between the groups (Table 2). Funnel plot asymmetry, along with significant Egger's and Begg's tests, suggested potential publication bias for HbA1c outcomes (Supplementary File 6). After adjustment using the trim-and-fill method, the estimated pooled effect size remained stable (WMD: −0.55%; 95% CI: −0.70 to −0.39; *P* < 0.001).

**Table 2:** Subgroup analyses for HbA1c.

Variables	Subgroup	No of studies [Reference]	WMD and 95%CI	*P* value	*I^2^* (%)	Q statistic	Interaction test
Country	Eastern	26 (27, 28, 34, 36, 38–42, 46–49, 51–53, 55, 57–60, 62, 66, 67, 71, 73)	−0.66 (−0.91 to −0.41)	< 0.001	98.3	< 0.001	< 0.001
	Western	20 (25, 26, 29–33, 37, 43–45, 50, 54, 56, 63–65, 69, 70, 72)	−0.42 (−0.70 to −0.13)	0.005	98.5	< 0.001	
Sample size	≤ 100	12 (25, 38, 43, 46, 47, 50, 54, 60, 63, 67, 69, 72)	−0.59 (−1.04 to −0.15)	0.009	92.0	< 0.001	< 0.001
	>100	34 (26–34, 36, 37, 39–42, 44, 45, 48, 49, 51–53, 55–59, 62, 64–66, 70, 71, 73)	−0.52 (−0.67 to −0.37)	< 0.001	98.4	< 0.001	
Mean age (years)	≤ 60	26 (27, 30, 32, 39–42, 44, 47, 49, 50, 52–58, 60, 62, 63, 66, 67, 69, 72, 73)	−0.65 (−0.89 to −0.41)	< 0.001	97.2	< 0.001	< 0.001
	>60	19 (25, 28, 29, 31, 33, 34, 36–38, 43, 45, 46, 48, 51, 59, 64, 65, 70, 71)	−0.38 (−0.54 to −0.21)	< 0.001	97.4	< 0.001	
Male (%)	≤ 60	34 (25, 27, 29–31, 33, 34, 36–38, 41, 42, 44, 45, 47, 48, 50–60, 62, 63, 66, 67, 69, 71, 72)	−0.63 (−0.84 to −0.43)	< 0.001	98.5	< 0.001	< 0.001
	>60	10 (28, 32, 39, 43, 46, 49, 64, 65, 70, 73)	−0.21 (−0.59 to 0.16)	0.264	98.3	< 0.001	
BMI (kg/m^2^)	≤ 30	17 (25, 27, 28, 38, 42, 43, 46, 47, 49, 53, 57–59, 62, 67, 70, 73)	−0.37 (−0.56 to −0.19)	< 0.001	95.0	< 0.001	< 0.001
	>30	15 (29–33, 37, 44, 45, 54, 56, 63–65, 69, 72)	−0.48 (−0.81 to −0.14)	0.005	98.9	< 0.001	
HbA1c (%)	≤ 8.0	16 (28, 29, 31, 37, 43–46, 50–52, 56, 60, 65, 66, 73)	−0.40 (−0.64 to −0.16)	0.001	98.8	< 0.001	< 0.001
	>8.0	25 (25, 27, 30, 32–34, 36, 38–42, 47, 49, 53–55, 57, 58, 62–64, 67, 71, 72)	−0.67 (−0.88 to −0.47)	< 0.001	95.5	< 0.001	
Delivery format	Face–to–face	24 (25–29, 31, 34, 36, 40–43, 45–47, 49, 53, 55, 58, 60, 67, 71, 73)	−0.51 (−0.70 to −0.33)	< 0.001	98.5	< 0.001	< 0.001
	Remote	10 (30, 32, 39, 54, 56, 63–65, 69, 70)	−0.50 (−0.75 to −0.26)	< 0.001	79.2	< 0.001	
	Blended	12 (33, 38, 44, 48, 50–52, 57, 59, 62, 66, 72)	−0.63 (−1.05 to −0.21)	0.003	97.8	< 0.001	
Session type	Group–based	16 (27, 29, 31, 37, 40, 42–44, 47, 50, 52, 58–60, 72, 73)	−0.48 (−0.84 to −0.13)	0.008	98.8	< 0.001	< 0.001
	Individual–based	30 (25, 26, 28, 30, 32–34, 36, 38, 39, 41, 45, 46, 48, 49, 51, 53–57, 62–67, 69–71)	−0.58 (−0.77 to −0.39)	< 0.001	97.0	< 0.001	
Theory–based intervention	Yes	19 (27, 30, 39, 40, 42, 43, 45, 47, 50–52, 55, 58, 60, 66, 70–73)	−0.58 (−0.88 to −0.29)	< 0.001	94.8	< 0.001	< 0.001
	No	27 (25, 26, 28, 29, 31–34, 36–38, 41, 44, 46, 48, 49, 53, 54, 56, 57, 59, 62–65, 67, 69)	−0.49 (−0.66 to −0.33)	< 0.001	98.5	< 0.001	
Multidisciplinary involvement	Yes	10 (26, 27, 41, 43, 48, 52, 58, 62, 63, 66)	−0.86 (−1.15 to −0.58)	< 0.001	83.8	< 0.001	< 0.001
	No	36 (25, 28–34, 36–40, 42, 44–47, 49–51, 53–57, 59, 60, 64, 65, 67, 69, 73)	−0.46 (−0.63 to −0.30)	< 0.001	98.7	< 0.001	
Follow–up (months)	≤ 6.0	30 (32–34, 36, 38, 42, 44, 46–50, 52–60, 62–64, 66, 67, 69, 70, 72, 73)	−0.64 (−0.88 to −0.40)	< 0.001	97.9	< 0.001	< 0.001
	>6.0	16 (25–31, 37, 39–41, 43, 45, 51, 65, 71)	−0.33 (−0.47 to −0.19)	< 0.001	93.8	< 0.001	

### FBG and 2hPG

**Figure 3:**
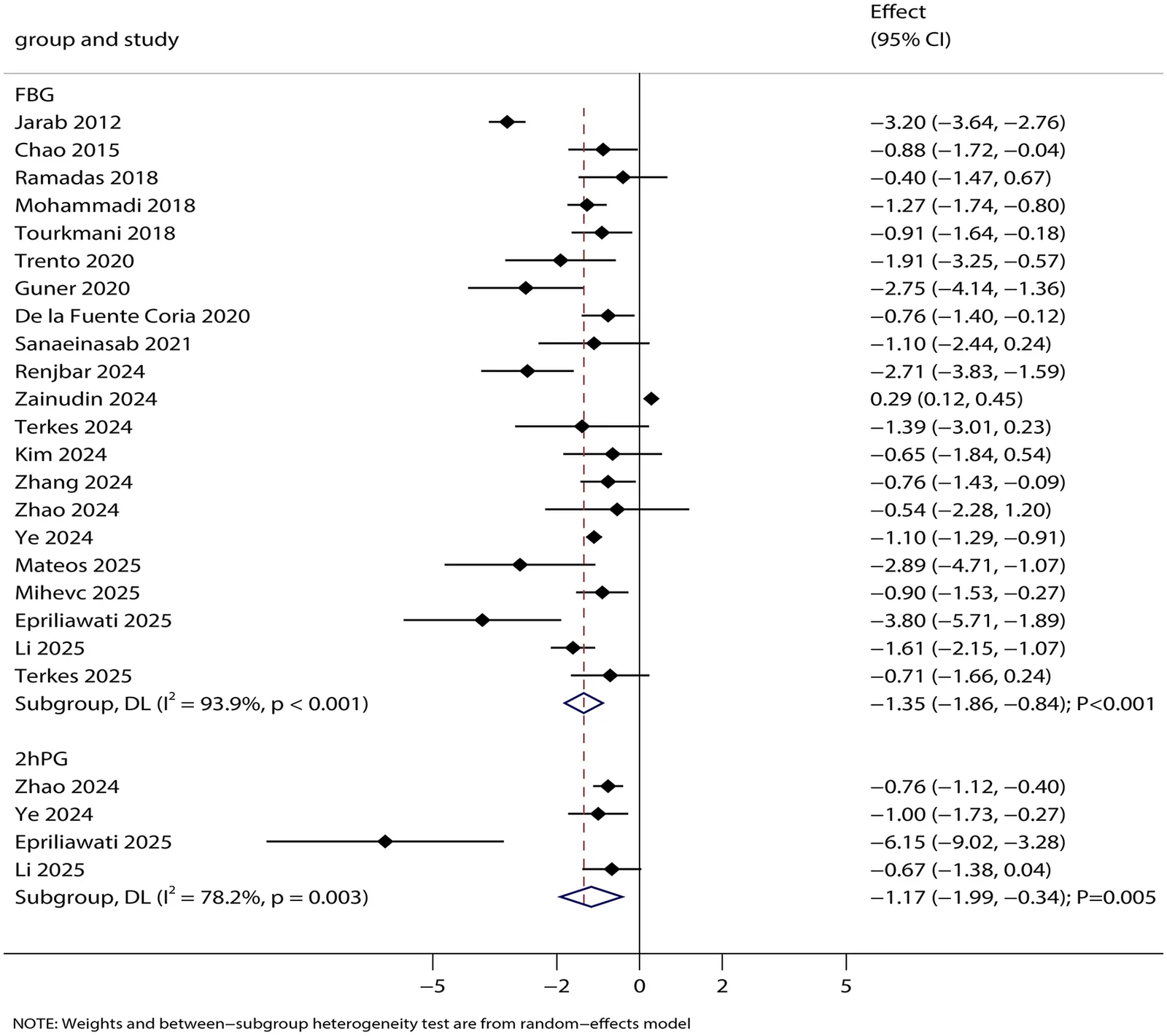
Forest plot showing the effect of structured diabetes education programs on fasting blood glucose (FBG) and 2–h postprandial glucose (2hPG).

A total of 21 studies reported post-intervention FBG outcomes. Compared with the control group, patients receiving education programs showed a significant reduction in FBG levels (WMD: −1.35 mmol/L; 95% CI: −1.86 to −0.84; *P* < 0.001; Figure 3). Considerable heterogeneity was observed (*I*^2^ = 93.9%, *p* < 0.001). Sensitivity analysis confirmed the robustness of the findings (Supplementary File 4). There was potential significant publication bias for FBG, and the conclusion was not affected after adjusting using the trim and fill method (Supplementary File 6).

A total of 4 studies reported post-intervention 2hPG outcomes. Education programs were associated with a significant reduction in 2hPG levels (WMD: −1.17 mmol/L; 95% CI: −1.99 to −0.34; *P* = 0.005), with high heterogeneity (*I*^2^ = 78.2%, *p* = 0.003). Sensitivity analysis indicated that the effect was not driven by any single study. The forest plot is presented in Supplementary File 6 and no publication bias was observed.

### BMI

**Figure 4:**
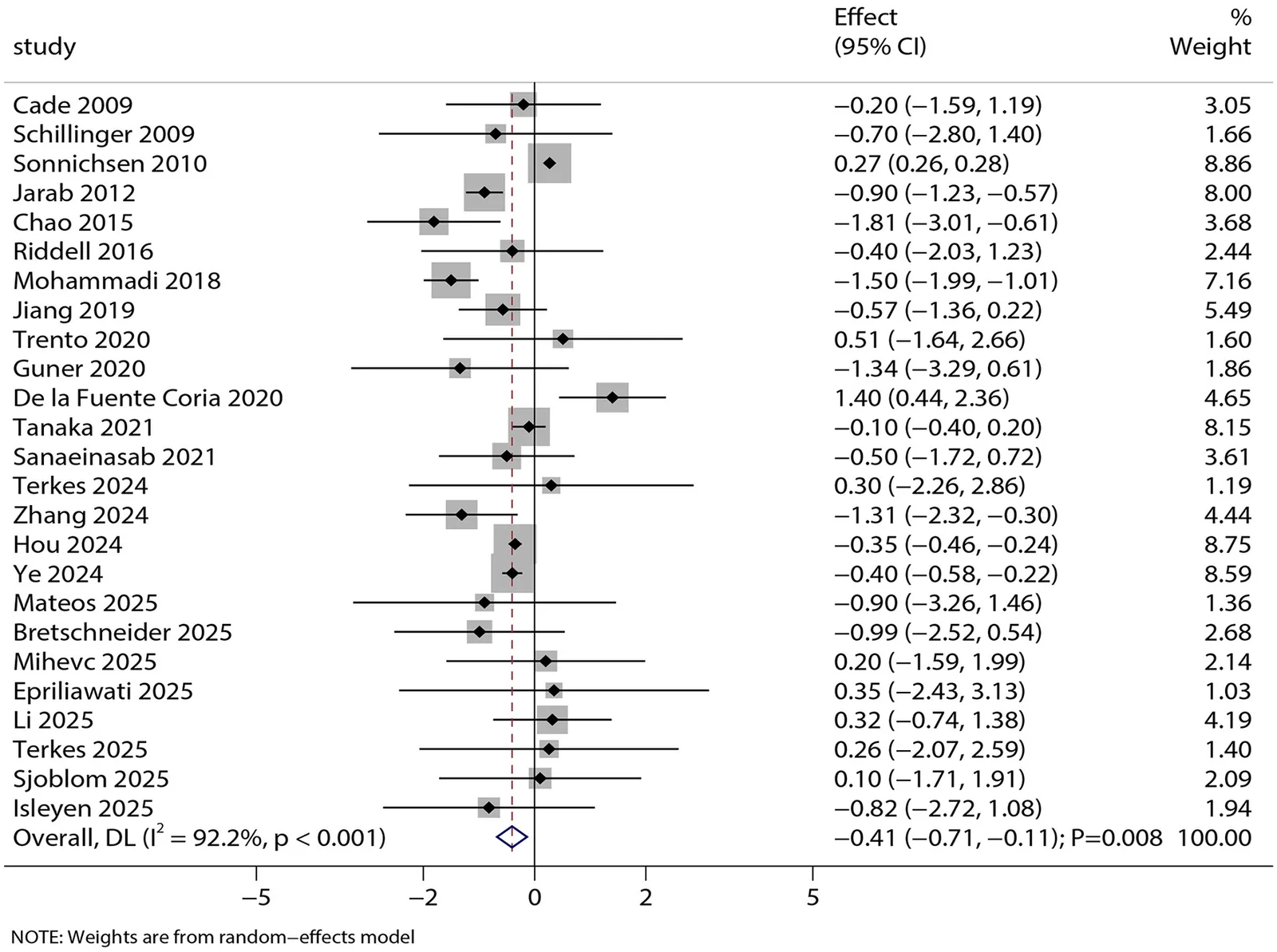
Forest plot showing the effect of structured diabetes education programs on body mass index (BMI).

A total of 25 studies reported post-intervention BMI outcomes. Education programs were associated with a modest but significant reduction in BMI compared with the control group (WMD = −0.41 kg/m^2^, 95% CI: −0.71 to −0.11; *P* = 0.008; Figure 4). Significant heterogeneity was present among the studies (*I*^2^ = 92.2%, *p* < 0.001). Sensitivity analysis indicated that the reduction remained statistically significant and was not influenced by any single study (Supplementary File 4). Publication bias was detected, but the adjusted pooled effect size remained stable after correction (WMD = −0.41 kg/m^2^, 95% CI: −0.71 to −0.11; *P* = 0.008; Supplementary File 6).

### SBP and DBP

**Figure 5:**
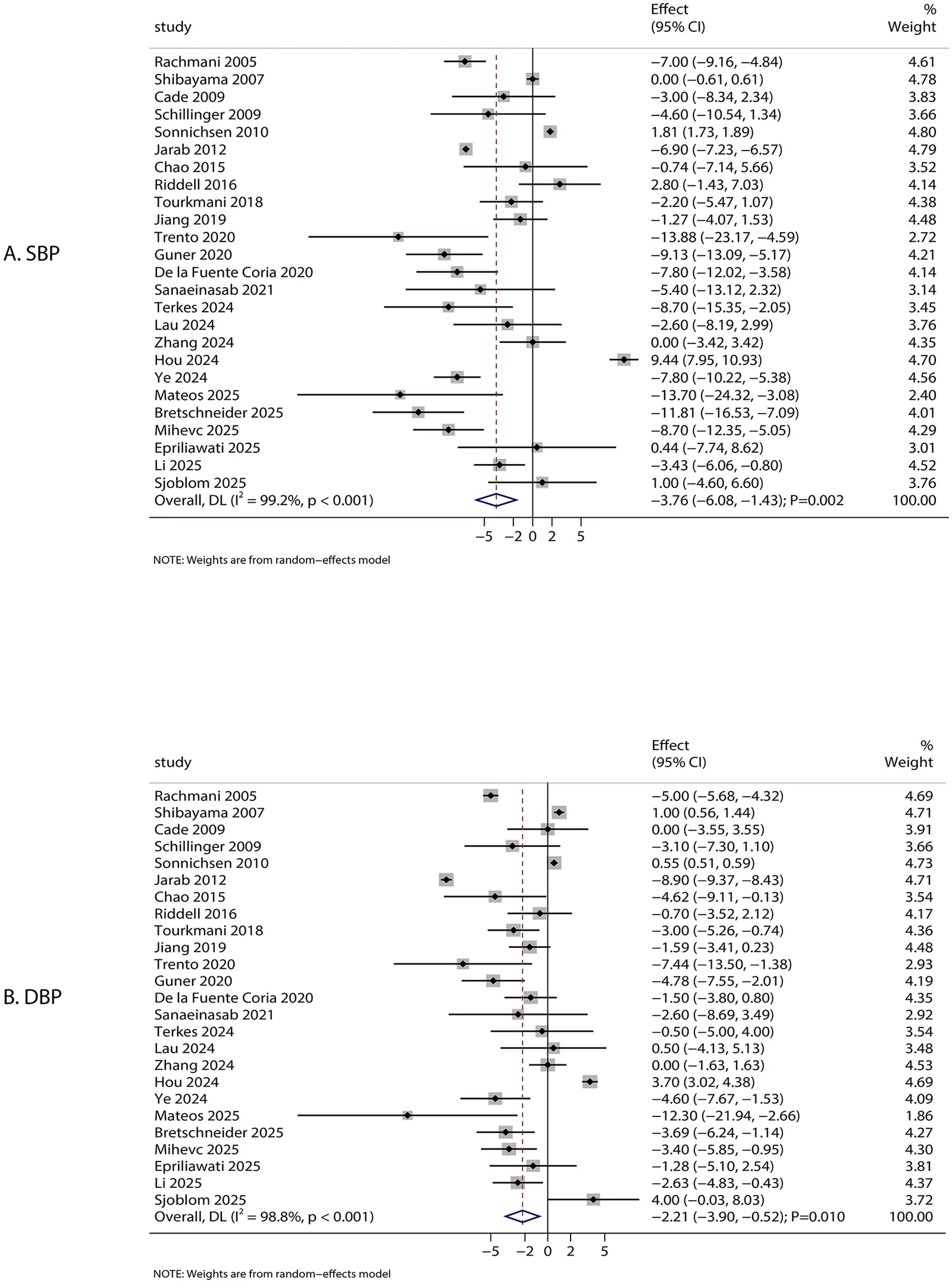
Forest plots showing the effect of structured diabetes education programs on blood pressure. **(A)** Systolic blood pressure (SBP). **(B)** Diastolic blood pressure (DBP).

A total of 25 studies reported outcomes for both SBP and DBP. Patients receiving education programs showed significant reductions in SBP (WMD: −3.76 mmHg; 95% CI: −6.08 to −1.43; *P* = 0.002) and DBP (WMD: −2.21 mmHg; 95% CI: −3.90 to −0.52; *P* = 0.010) (Figure 5). Significant heterogeneity was observed for both SBP (*I*^2^ = 99.2%, *p* < 0.001) and DBP (*I*^2^ = 98.8%, *p* < 0.001). Sensitivity analysis confirmed that the pooled effect sizes for both outcomes remained statistically significant after excluding any single study (Supplementary File 4). No significant publication bias was detected for either SBP or DBP outcomes (Supplementary File 6).

### Lipid profiles

**Figure 6:**
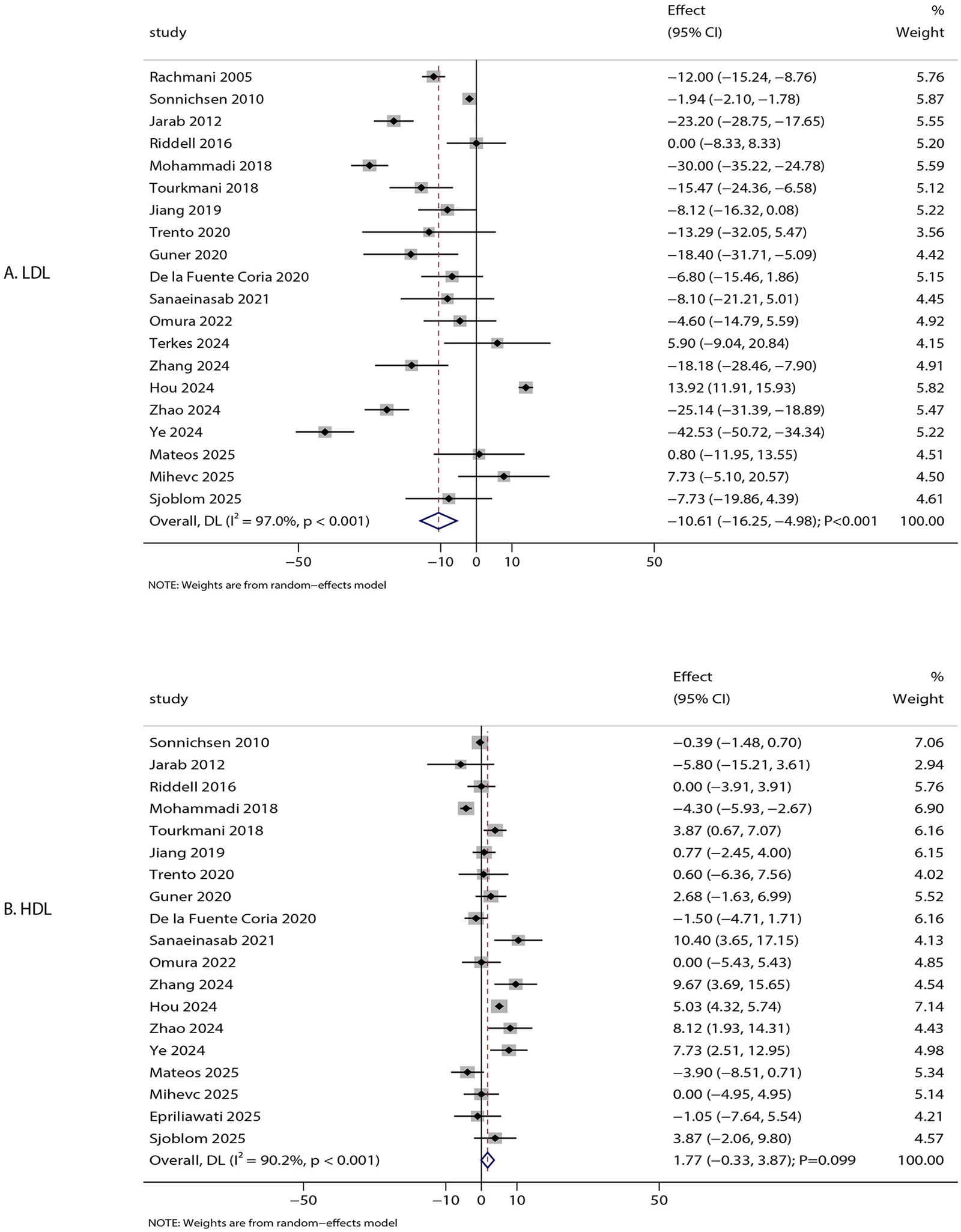
Forest plots showing the effect of structured diabetes education programs on lipid profiles (Part I). **(A)** Low–density lipoprotein cholesterol (LDL–C). **(B)** High–density lipoprotein cholesterol (HDL–C).

LDL-C: A total of 20 studies reported post-intervention LDL-C levels. Education programs were associated with a significant reduction in LDL-C (WMD: −10.61 mg/dL; 95% CI: −16.25 to −4.98; *P* < 0.001; Figure 6), with significant heterogeneity (*I*^2^ = 97.0%, *p* < 0.001). Sensitivity analysis indicated that the reduction was not driven by any single study (Supplementary File 4). No significant publication bias was detected (Supplementary File 6).

HDL-C: A total of 19 studies reported post-intervention HDL-C levels. No significant difference was observed between the education and control groups (WMD: 1.77 mg/dL; 95% CI: −0.33 to 3.87; *P* = 0.099; Figure 6). Significant heterogeneity was present (*I*^2^ = 90.2%, *p* < 0.001). Sensitivity analysis suggested a potential increase in HDL-C in the education group (Supplementary File 4). No significant publication bias was detected (Supplementary File 6).

**Figure 7:**
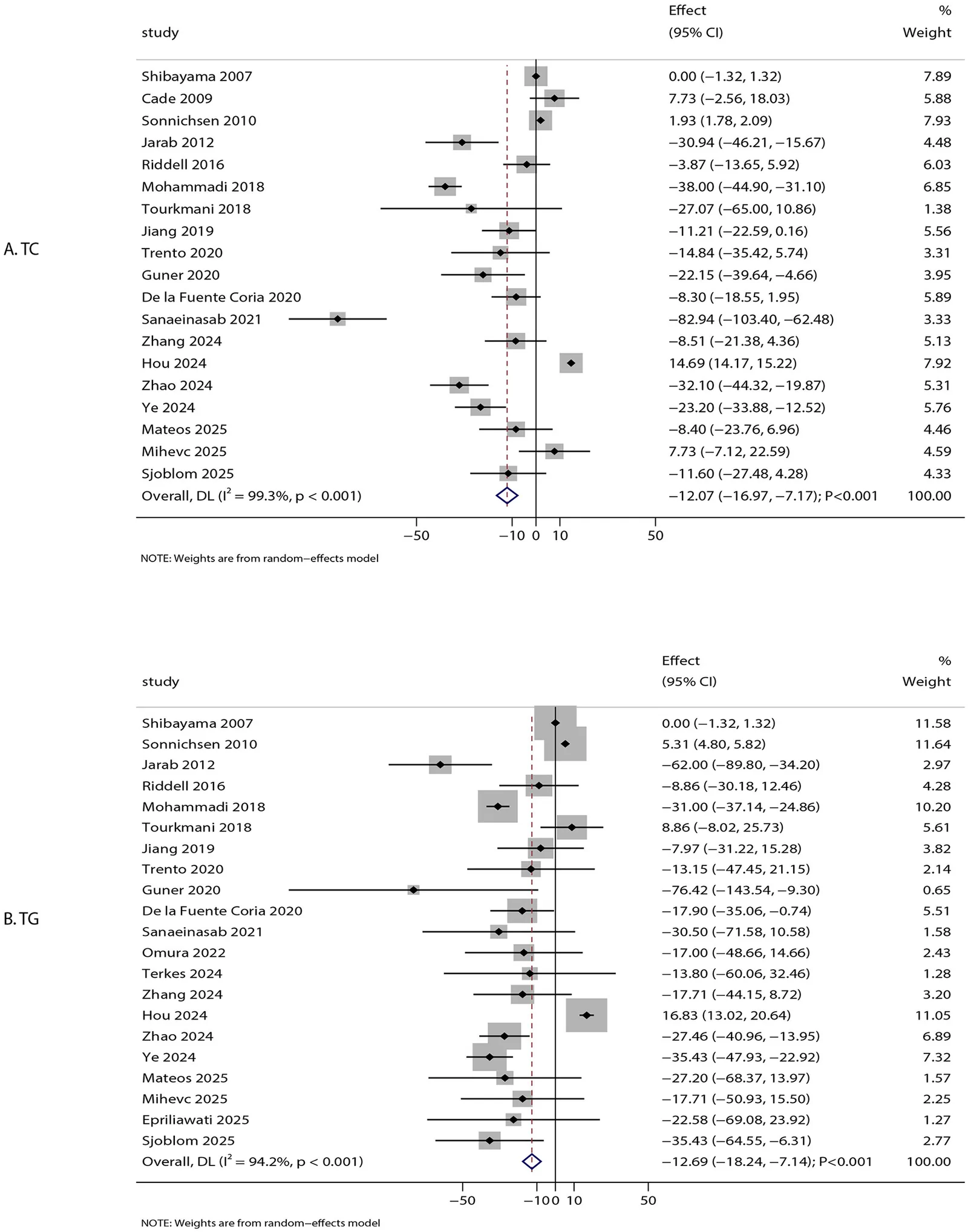
Forest plots showing the effect of structured diabetes education programs on lipid profiles (Part II). **(A)** Total cholesterol (TC). **(B)** Triglycerides (TG).

TC: A total of 19 studies reported post-intervention TC levels. Education programs were associated with a significant reduction in TC (WMD: −12.07 mg/dL; 95% CI: −16.97 to −7.17; *P* < 0.001; Figure 7), with significant heterogeneity (*I*^2^ = 99.3%, *p* < 0.001). Sensitivity analysis indicated that the reduction was robust and not influenced by any single study (Supplementary File 4). No significant publication bias was detected (Supplementary File 6).

TG: A total of 21 studies reported post-intervention TG levels. Education programs were associated with a significant reduction in TG (WMD: −12.69 mg/dL; 95% CI: −18.24 to −7.14; *P* < 0.001; Figure 7), with significant heterogeneity (*I*^2^ = 94.2%, *p* < 0.001). Sensitivity analysis indicated that the reduction was not driven by any single study (Supplementary File 4). Funnel plot asymmetry and Egger's test suggested potential publication bias for TG outcomes (Supplementary File 6). After adjustment using the trim-and-fill method, the estimated pooled effect size remained stable (WMD: −12.69 mg/dL; 95% CI: −18.24 to −7.14; *P* < 0.001).

## Discussion

This systematic review and meta-analysis, encompassing 49 RCTs with a total of 9,587 patients with T2D, provides the most up-to-date and comprehensively synthesis of the effects of structured education programs on multiple health outcomes. Our primary findings indicate that, compared to usual care, participation in education programs was associated with statistically significant improvements in glycemic control (mean HbA1c reduction: 0.55%; FBG reduction: −1.35 mmol/L; 2hPG: −1.17 mmol/L), weight management (mean BMI reduction: 0.41 kg/m^2^), blood pressure control (mean reductions: SBP −3.76 mmHg, DBP −2.21 mmHg), and lipid profiles (LDL-C: −10.61 mg/dL, TC: −12.07 mg/dL, TG: −12.69 mg/dL). However, the substantial heterogeneity observed across nearly all outcomes underscores the need to understand which intervention characteristics and patient factors drive the variability in treatment effects—a focus that distinguishes our review from previous syntheses.

First, the effect size observed for HbA1c reduction (WMD −0.55%) is clinically significant. This magnitude of reduction is comparable to the efficacy of certain first-line oral anti-diabetic agents as monotherapy (74–76). It is well-established that a 1% reduction in HbA1c significantly decreases the risk of diabetes-related microvascular complications (77). Therefore, integrating high-quality patient education into standard treatment may not only directly improve glycemic levels but also confer long-term health benefits. However, our analysis revealed substantial heterogeneity (I2 = 98.6%), suggesting considerable variability in intervention effects across studies. Our meta-regression analyses provide important insights into the sources of this heterogeneity and the factors that moderate intervention effectiveness. Notably, the proportion of males was significantly and inversely associated with the magnitude of HbA1c reduction, confirming and extending our subgroup finding that educational interventions may be less effective in male-dominated populations. This finding aligns with previous research suggesting gender differences in health behavior modification, illness perception, and access to social support (78, 79). Future program design may need to incorporate greater gender sensitivity, such as male-specific peer support groups, communication strategies that appeal to male preferences, or interventions addressing masculine norms that may conflict with diabetes self-management. Multidisciplinary team involvement was significantly associated with larger effect sizes, suggesting that comprehensive education programs delivered by teams of healthcare professionals (e.g., nurses, physicians, dietitians, pharmacists, and health educators) may be more effective than those delivered by single providers. Multidisciplinary teams can address multiple aspects of diabetes self-management simultaneously, providing integrated care that addresses medical, nutritional, psychological, and behavioral needs. In addition, the significant reductions in FBG and 2hPG further support the beneficial effects of education programs on glycemic control. While HbA1c remains the gold standard for long-term glycemic assessment, these fasting and postprandial glucose improvements suggest that education programs may influence both baseline glucose regulation and postprandial glycemic excursions. The consistent direction of effects across all three glycemic measures (HbA1c, FBG, and 2hPG) strengthens the overall evidence that structured education programs effectively improve glycemic control through multiple pathways.

Second, this study confirmed the positive impact of education programs on cardiometabolic risk factors (BMI, blood pressure, lipids). Although the reduction in BMI (−0.41 kg/m^2^) appears modest, it holds public health significance at the population level, and weight management remains a cornerstone of T2D care. The reductions in blood pressure and LDL-C are particularly crucial, as cardiovascular disease is the leading cause of mortality in T2D patients (80, 81). The ability of education programs to concurrently improve multiple cardiovascular risk markers suggests their mechanism of action extends beyond mere “knowledge transfer,” likely operating through the promotion of comprehensive healthy lifestyle changes (82). It is noteworthy that the effect on increasing HDL-C levels was not significant, which may be attributed to the stronger genetic influence on HDL-C or the requirement for longer-term lifestyle intervention to induce changes.

Our findings are largely consistent with major reviews published in recent years but offer broader global evidence and more updated data support (12–14). This analysis further extends current understanding by highlighting the potential of education programs to improve composite cardiometabolic endpoints. The observed high heterogeneity also serves as a reminder that not all education programs are equally effective. Variations in outcomes may stem from multiple factors, including the evidence-based nature of the content, intervention intensity, professional background of the implementers, cultural adaptation, and participant characteristics. Future research should aim to identify and validate the “active components” within education programs that are most effective for specific outcomes.

To examine whether intervention effects were consistent across outcomes, we assessed whether studies showing the strongest HbA1c improvements also demonstrated stronger effects on other cardiometabolic outcomes. Among studies that reported both HbA1c and at least two other outcomes, we observed a general trend toward consistency: studies with the largest HbA1c reductions (WMD < −0.80%) (26, 33, 41, 42, 44, 62, 67) also tended to show larger reductions in SBP (range: 0.44 to −9.13 mmHg), DBP (range: −1.28 to −8.90 mmHg), and LDL-C (range: −8.12 to −42.53 mg/dL). Conversely, studies with smaller HbA1c effects (WMD > −0.20%) generally showed smaller or non–significant improvements in other outcomes. However, this trend was not universal: some studies with modest HbA1c improvements still demonstrated substantial blood pressure or lipid improvements (58, 67), suggesting that the effects on different outcomes may be partially independent and influenced by specific intervention components targeting different behaviors. Our cross–outcome consistency analysis revealed that studies with larger HbA1c reductions generally also showed larger improvements in other cardiometabolic outcomes, supporting the overall effectiveness of comprehensive, intensive education programs. However, the observation that some studies with modest HbA1c effects still achieved substantial blood pressure or lipid improvements suggests that education programs may have outcome–specific mechanisms of action. This finding has important implications for program design: interventions may need to be tailored to target specific risk factors based on individual patient needs, rather than assuming a “one–size–fits–all” approach.

To further explore potential drivers of the observed heterogeneity, we compared the intervention characteristics of studies with the largest (WMD < −1.0%) and smallest (WMD > 0%) effects on HbA1c reduction. Studies with larger effects tended to employ more intensive interventions, including multiple face–to–face sessions (typically >6 sessions) (52, 67), longer intervention durations (≥6 months) (36, 41, 52, 62, 67, 71), and multidisciplinary delivery teams (including nurses, pharmacists, dietitians, and physicians) (41). In contrast, studies with smaller or non–significant effects often involved less intensive interventions, such as brief counseling (50), or predominantly self–directed digital interventions without active follow–up (39, 65). These observations suggest that intervention intensity, delivery mode, multidisciplinary involvement, and theoretical grounding may be key factors influencing the effectiveness of educational programs.

Our findings extend and refine the evidence base established by previous systematic reviews in several important ways. First, while Versluis et al. (12) reported that mHealth interventions improved HbA1c by −0.30% based on studies published up to 2023, our updated analysis incorporating newer trials suggests that the effect of digital interventions may be somewhat larger (−0.55%), potentially reflecting the inclusion of less intensive or less interactive programs in the more recent literature. Second, unlike Colaone et al. (13), who examined telemedicine interventions for weight management, our review demonstrates that digital and remote interventions—while generally effective—may produce smaller effects than face–to–face programs. Third, compared to Sun et al. (14), who focused specifically on nurse–led education and reported an HbA1c reduction based on 8 studies, our broader inclusion of diverse delivery formats yielded a slightlysmaller effect size and confirmed that the benefits extend beyond nurse–delivered programs. Fourth, our review provides a more comprehensive assessment of cardiometabolic outcomes than most previous reviews, confirming significant improvements in multiple cardiovascular risk factors. Notably, our finding that theory–based interventions yielded significantly larger effects than those without explicit theoretical grounding is a novel contribution that was not systematically examined in earlier reviews.

Based on our findings, we offer the following recommendations for clinical practice: (1) Integration into standard care: structured education programs should be integrated as a core component of standard T2D care, not merely an optional add–on. Our findings support the inclusion of patient education in clinical practice guidelines and quality assessment frameworks; (2) Targeting high–risk patients: given that patients with higher baseline HbA1c showed greater absolute improvements, resources for education programs should be prioritized for patients with poorly controlled diabetes; (3) Multidisciplinary team involvement: our meta–regression analysis revealed that multidisciplinary team involvement was significantly associated with larger effect sizes. Comprehensive programs addressing multiple aspects of self–management (diet, exercise, medication, glucose monitoring) may be more effective than narrowly focused interventions; (4) Gender–sensitive approaches: uur meta–regression analysis identified proportion of males as a significant moderator, with smaller effects in male–dominated populations. Clinicians should consider gender–specific barriers and preferences in program design, such as male–specific peer support groups, communication strategies that appeal to male preferences, or interventions addressing masculine norms that may conflict with diabetes self–managemen; (5) Cultural adaptation: while country region was not a significant moderator, the variation in effect sizes across studies suggests that cultural adaptation of interventions is important. Programs should be tailored to the cultural context, including language, values, beliefs, and health literacy levels; and (6) Technology integration: given the effectiveness of remote/blended interventions, healthcare systems should consider integrating digital health tools (web–based platforms, mobile apps, SMS reminders, telehealth) into diabetes education programs. Therefore, recommendations for future research: (1) head–to–head comparisons: future studies should conduct head–to–head comparisons of different education program components to identify the ‘active ingredients' that drive effectiveness. Randomized controlled trials with factorial designs could address this question; (2) long–term follow–up: studies with longer follow–up periods (≥2 years) are needed to assess the sustainability of benefits and determine whether booster sessions are needed to maintain improvements; (3) cost–effectiveness analyses: formal cost–effectiveness analyses of education programs compared to standard care would inform resource allocation decisions; (4) patient–reported outcomes: future studies should include patient–reported outcomes such as quality of life, diabetes distress, self–efficacy, and treatment satisfaction to capture the full benefits of education programs; (5) implementation science research: research is needed on effective strategies for implementing evidence–based education programs in diverse healthcare settings, including primary care, community health centers, and low–resource settings; (6) digital health integration: given the growing role of digital health, future studies should examine the optimal integration of digital and in–person components to maximize effectiveness while maintaining cost–efficiency; (7) individual participant data (IPD) meta–analysis: an IPD meta–analysis would enable exploration of patient–level moderators (e.g., socioeconomic status, health literacy, comorbidities, medication use) that cannot be examined in aggregate data meta–analyses; and (8) mechanistic studies: research is needed to understand the mechanisms through which education programs achieve their effects, including the role of self–efficacy, health beliefs, social support, and behavioral change processes.

This study has several limitations. First, despite employing meta–regression to explore sources of heterogeneity, majority of the between–study variance remained unexplained, suggesting that additional unmeasured factors—such as intervention quality, participant engagement, cultural adaptation, and healthcare system characteristics—may influence treatment effects. The substantial variation in intervention approaches across studies means that the interventions may not be directly comparable. Second, the follow–up duration in most studies was less than one year, leaving the long–term sustainability of the educational effects uncertain. Third, although publication bias was assessed and adjusted for, some outcomes may still be susceptible to undetected negative studies. Fourth, the description of program content and delivery format varied in detail across studies, limiting a more in–depth comparative analysis of their core mechanisms. Finally, the analysis was based primarily on aggregate data, precluding the exploration of individual–level factors moderating the educational effect.

## Conclusion

This systematic review and meta–analysis provides robust evidence that structured education programs for patients with T2D are an effective intervention strategy, leading to clinically meaningful improvements in glycemic control and conferring broad cardiometabolic benefits that, when considered collectively, may substantially reduce overall cardiovascular risk. These findings strongly support the integration of standardized, high–quality patient self–management education and support as a fundamental component of standard T2D care, and its inclusion in clinical practice guidelines and healthcare quality assessment frameworks. Clinicians, healthcare administrators, and policymakers should prioritize the implementation of theory–based, multidisciplinary, and culturally adapted education programs of sufficient intensity to achieve meaningful improvements in patient outcomes.

## Data Availability

The original contributions presented in the study are included in the article/Supplementary material, further inquiries can be directed to the corresponding author.
