# Supplementary material for: Effect of educational program for patients with type 2 diabetes: a systematic review and meta-analysis of randomized controlled trials
Source: Front Med (Lausanne). 2026 Aug 13;13:1860805. doi: 10.3389/fmed.2026.1860805 (PMC13518541; doi:10.3389/fmed.2026.1860805)
Supplement: Supplementary file 1 [file Table_1.docx]

**PubMed:**

("Health Education"[Mesh] OR "Patient Education as Topic"[Mesh] OR "Consumer Health Information"[Mesh]) OR ("health education"[tiab] OR "patient education"[tiab] OR psychoeducation[tiab] OR "therapeutic education"[tiab] OR "consumer health information"[tiab] OR "health knowledge"[tiab] OR "client education"[tiab])

AND

("Diabetes Mellitus, Type 2"[Mesh] OR "type 2 diabetes"[tiab] OR T2DM[tiab] OR "non insulin dependent diabetes"[tiab] OR NIDDM[tiab])

**EmBase:**

1. exp health education/ or exp patient education/ or exp psychoeducation/ or health promotion/

2. (health education or patient education or psychoeducation or therapeutic education or consumer health information or health knowledge or client education).ti,ab,kw.

3. 1 or 2

4. exp non insulin dependent diabetes mellitus/

5. (type 2 diabetes or T2DM or NIDDM or noninsulin dependent diabetes).ti,ab,kw.

6. 4 or 5

7. 3 and 6

**Cochrane Library:**

1. “Health Education” OR “Patient Education as Topic” OR “Consumer Health Information”
2. “health education” OR “patient education” OR psychoeducation OR “therapeutic education” OR “consumer health information” OR “health knowledge” OR “client education”
3. #1 OR #2
4. “Diabetes Mellitus, Type 2”
5. “type 2 diabetes” OR T2DM OR “non insulin dependent diabetes” OR NIDDM
6. #4 OR #5
7. #3 AND #6

**Web of Science:**

TS=(("health education" OR "patient education" OR psychoeducation OR "therapeutic education" OR "consumer health information" OR "health knowledge" OR "client education")) AND TS=(("type 2 diabetes" OR T2DM OR "non insulin dependent diabetes" OR NIDDM))
