# Supplementary material for: Effect of educational program for patients with type 2 diabetes: a systematic review and meta-analysis of randomized controlled trials
Source: Front Med (Lausanne). 2026 Aug 13;13:1860805. doi: 10.3389/fmed.2026.1860805 (PMC13518541; doi:10.3389/fmed.2026.1860805)
Supplement: Supplementary file 2 [file Table_2.docx]

Table S1. Comprehensive intervention characteristics for all 49 studies

| Trial | Delivery Mode | Interventionists | Theory-based | Additional Techniques | Frequency | Session Duration | Total Educational Sessions | Education Content |
| --- | --- | --- | --- | --- | --- | --- | --- | --- |
| Goudswaard 2004 [25] | Face-to-face; individual-based | Two skilled diabetes nurses | No | None specified | Six sessions over 6 months, at intervals of 3-6 weeks | 15-45 minutes per session (total contact time approximately 2.5 hours) | 6 sessions | General information on diabetes; reinforcing compliance with medication; importance of physical exercise and losing body weight; nutritional advice; self-monitoring of blood glucose |
| Rachmani 2005 [26] | Face-to-face; individual-based | Consultation team members | No | Encouraged patients to measure BP weekly; keep records of laboratory results; urge physicians to change/intensify treatment if target values not reached; encouraged to call consultants when advice needed; individualized plan of lifestyle modification and fitness program | Two initial 2-hour teaching sessions; thereafter patients initiated additional consultations | 2 hours per teaching session | 2 initial teaching sessions | Ways to achieve tight control of modifiable risk factors (BP, LDL cholesterol, HbA1c); individualized lifestyle modification plan; fitness program; self-monitoring of BP; how to track laboratory results; how to communicate with physicians to intensify treatment |
| Ko 2007 [27] | Face-to-face; group-based; inpatient setting | Multidisciplinary team: diabetologist, certified diabetes educator, ophthalmologist, rehabilitation therapist, pharmacist, psychologist, family medicine doctor, and rehabilitation medicine doctor | Yes | Group discussion sessions; family members invited to attend; annual reinforcement education with review of self-management and new topics; telephone or e-mail contact if patients did not attend scheduled visits | Inpatient: 5 consecutive days (6 hours/day); Outpatient follow-up: every 3 months; Annual reinforcement: single session per year | Approximately 6 hours per day for 5 days (total 30 hours inpatient) + 3 hours annual reinforcement | 5 full days of inpatient education (30 hours total) + annual reinforcement sessions (3 hours each) | Understanding of diabetes itself; self-glucose monitoring using a meter; injection techniques; sick day care; meal planning (based on DPP and Food Pyramid guidelines: total fat ≤30% energy, saturated fat 8–10%, monounsaturated ≤10%, polyunsaturated ≤15%, cholesterol <300 mg/day, desirable body weight maintenance); physical activity (DPP guidelines); foot inspection; management of hypoglycaemia; stress management; group discussions; foot clinic; retinal examination; laboratory examination |
| Shibayama 2007 [28] | Face-to-face; individual-based | Certified Expert Nurse (CEN) in diabetes nursing | No | Attentive listening; empathy; patient participation in goal setting; selecting personalized strategies to overcome barriers; follow-up including evaluation and problem solving; provision of educational materials of CEN's own making and printed laboratory results if necessary | Monthly at hospital visits for 1 year | 8-76 minutes (median, 25 minutes) | Approximately 12 sessions over 1 year (monthly) | Assessment of eating patterns, level of physical activity, adherence to medication, level of self-care for diabetic complications, management of daily stress; identification of most problematic areas and patient's barriers to lifestyle changes; personalized goal setting for lifestyle change; specific intervention strategies to decrease barriers and empower patient |
| Cade 2009 [29] | Face-to-face; group-based; lay-led | Lay peer educators + one senior trainer from NHS EPP. Four peer tutors delivered the courses. All tutors received additional training to deliver diabetes-specific elements (week 7) | No | None specified beyond the standardized EPP format (problem solving, relaxation techniques, peer support, goal setting) | Once weekly for 7 weeks | 2 hours per session | 7 sessions (6 standard EPP sessions + 1 additional diabetes-specific session) | First 6 sessions (standard EPP): Learning to cope with long-term health problems; improved eating patterns; relaxation techniques; exercise patterns; self-management skills including problem solving; goal setting. Week 7 (diabetes-specific session): Identifying common problems for people with diabetes; monitoring diabetes; self-managing diabetes in terms of food intake, physical activity, blood glucose, and blood pressure; goal setting |
| Schillinger 2009 [30] | Remote; individual-based; technology-mediated with interpersonal nurse follow-up as needed | Automated telephone system + nurse care managers | Yes | Prerecorded automated calls; patient responses triggered immediate automated health education messages and/or subsequent nurse phone follow-up; action plans; hierarchical logic; proactive communication; tailored to English, Spanish, and Cantonese | Weekly automated calls over 39 weeks (9 months) | 6–10 minutes per call for patients to complete | Up to 39 automated calls; mean number completed among ever users was 21.9 calls; mean number of nurse call-backs was 9.2 | Diabetes self-management support; behavioral action plans (primarily exercise and/or diet); surveillance and monitoring; patient activation; collaborative goal-setting; skills enhancement; access to resources; tailored health education messages based on patient responses |
| Sonnichsen 2010 [31] | Face-to-face; group-based; conducted in GP surgeries or out-patient clinics | General practitioners (GPs) and primary care physicians who completed mandatory 10-hour face-to-face training course; patient education conducted by physicians in their surgeries or in out-patient clinics | No | Standardised documentation of physical examination, laboratory findings, and diabetes complications in DMP-form once a year; structured interdisciplinary care according to Austrian Diabetes Association guidelines; agreement on therapeutic goals in shared patient-physician decision-making process at three-monthly intervals; patient reminders; continuous feedback | Patient education: 4 modules; Therapeutic goal agreement: every 3 months; Standardised documentation: annually | Physician training: 10 hours total; Patient education: 9 hours total (4 modules, group size 3-12 patients) | Physician training: 1 course (10 hours); Patient education: 4 modules (9 hours total) | Physician training: Update in diabetes care; current guidelines of Austrian Diabetes Association (ÖDG); practice management training. Patient education (Düsseldorfer Modell curriculum): Diabetes self-management; disease knowledge; lifestyle modification; goal setting. Standard DMP components: Structured interdisciplinary care according to ÖDG guidelines; standardized documentation; agreement on therapeutic goals |
| Williams 2012 [32] | Remote (automated interactive telephone); individual-based; landline or mobile phone | Automated interactive telephone system developed collaboratively by Australian research team and researchers at Medical Information Systems Unit, Boston University, USA; TLC Coordinator provided brief telephone support for technical issues | No | Tailored feedback and encouragement based on information entered in TLC database and participant responses during calls; blood glucose upload via Bluetooth™ device; email "alerts" to study email address if unusual clinical issues arose; quarterly newsletter containing general health information sent to all participants (both arms); smoking cessation information pack provided for current smokers | Weekly calls requested over 6 months | 5–20 minutes per call depending upon call content and participant responses | Up to 24-26 weekly calls over 6 months; mean number of completed calls was 18 (± 6), range 2-27; mean percentage of completed calls out of expected weekly calls was 76% (± 22) | Blood glucose testing (first topic in each call); nutrition/healthy eating; physical activity; medication-taking; smoking cessation (information pack only). Clinical targets (recommended number of weekly blood glucose tests, blood glucose range, clearance for physical activity) obtained from participants' primary healthcare providers before first call. Self-care behaviours targeted: blood glucose testing, nutrition, physical activity, medication-taking |
| Jarab 2012 [33] | Face-to-face (baseline) + telephone (follow-up); individual-based; blended format | Clinical pharmacist | No | Motivational interviewing; objective-directed education; personalized discussion of prescribed therapy; telephone follow-up calls (8 weekly calls); special booklet on diabetes medications and lifestyle changes provided to patients; pharmacist recommendations discussed with physician when necessary; smoking cessation referral program within hospital; diabetes-specific biomarker targets specified for each patient | One face-to-face session at baseline + 8 weekly telephone follow-up calls | Face-to-face: duration not specified; Telephone calls: average 20 minutes each | 1 face-to-face session + 8 telephone calls = 9 total contacts | Type 2 diabetes disease information; risks for and types of complications; prescribed drug therapy, proper dosage, possible side effects; importance of medication adherence; lifestyle management (healthy dietary habits, regular physical activity, blood glucose monitoring and recording); smoking cessation advice (motivational interviewing); biomarker targets (A1c, BP, LDL). Specific booklet on diabetes medications and necessary lifestyle changes provided |
| Chow 2015 [34] | Face-to-face (home visits) + telephone; individual-based; home-based setting | Trained pharmacist | No | Home visits; two educational sessions per visit; reinforcement at second visit; information booklet on T2DM and food pyramid chart (Ministry of Health Malaysia); medication charts and pictorial labels for each medication for patients with difficulties identifying medications; telephone contact between visits to remind patients of monthly prescription refills and reassess medication use | Two home visits over 3 months + telephone calls between visits | 62.47 ± 5.24 minutes per home visit | 2 home visits + telephone follow-up calls | Session 1 (Medications): Proper use of medications; medication indications; dosage; administration frequencies; side effects and side effect management; storage and disposal of medications; importance of medication adherence. Session 2 (T2DM): Pathophysiology; risk factors; types of diabetes; signs and symptoms; diabetes complications; meal planning; lifestyle modifications; self-care. Reinforcement: Information from first visit repeated. Written materials: T2DM information booklet; food pyramid chart (MyHealth, Ministry of Health Malaysia); medication charts; pictorial medication labels |
| Chao 2015 [35] | Face-to-face + telephone; individual-based + group-based (blended: individual consultation and group lectures); community health service center setting | Specially-trained community health service center staff, managers, and related researchers | No | Health record establishment; health evaluation using specialized software; individual telephone consultation; group lectures; follow-up visits; distribution of health promoting materials; interventions administered at least once per month; monitoring of intervention compliance | At least once per month for 18 months | Not specified | At least 18 sessions over 18 months | Diet advice: Individualized dietary guidance. Psychological aspects of health: Individual psychological counseling/support. Exercise program: Tailor-made exercise program based on evaluation. Education/skills training on health self-management: Self-management skills training. Group lectures: Prevention and treatment of diabetic foot disease, cardio-cerebrovascular complications, diabetic nephropathy; rational lifestyle; self-management. Regular blood glucose monitoring: Encouragement and monitoring of regular glucose testing. Long-term diabetes drug monitoring: Medication adherence monitoring. Health promoting materials distribution: Educational materials provided |
| AI-Haj Mohd 2016 [36] | Face-to-face + telephone; individual-based; clinic setting | Diabetes nurse | No | Weekly telephone calls for 3 months; PowerPoint presentation; collaborative approach encouraging patients to ask questions; educational session on medication adherence | One 30-minute educational session + weekly telephone calls for 3 months + follow-up at 6 months | 30 minutes (educational session); telephone calls: not specified | 1 face-to-face session + 12 weekly telephone calls (approximately) | Importance of medication adherence; when and how to take medicine; benefits of medicine; what to expect in terms of side effects; diabetes disease information; medication action, timing, and benefits; barriers to self-management; patients encouraged to ask questions about medication adherence |
| Riddell 2016 [37] | Face-to-face; group-based (community-based meetings); lay-led | Lay peer supporters | No | Monthly community-based group meetings; goal setting and monitoring; problem solving; bi-monthly newsletter; peer supporter training (2.5 days); weekly teleconferences for peer supporters; peer supporter handbook; encouragement to arrange visits by local health professionals; encouragement of shared activities outside meetings (walks, tai chi, social gatherings); regular phone contact between supporters and participants | Monthly group meetings for 12 months + bi-monthly newsletters + regular phone contact + additional shared activities | 90 minutes per group meeting | 12 monthly group meetings (minimum) + additional activities + newsletters | Core functions: Assistance with daily self-management; provision of social and emotional support; promotion and support of regular linkage to clinical care; provision of ongoing and sustained support. Meeting topics: Key diabetes self-management behaviours; goal setting; problem solving; clinical targets; use of available information resources; visits by local health professionals. All participants also received: 7-hour DSME course + comprehensive manual. |
| Chen 2016 [38] | Face-to-face + telephone; individual-based; clinic setting | Certified diabetes-educator pharmacist | No | Assessment of adherence to pillbox use and insulin injection technique; appropriateness of current medication regimens; identification and resolution of drug-related problems; MMSE for cognitive assessment; TGDS-S for depression screening; individual diabetes education; recommendations to physicians; referral to CDE nurse and dietitians; monthly telephone calls for medication counseling and adverse event surveys; personal visits as needed; standardized screening tools for cognitive function and depression | At least 1 face-to-face visit at enrollment + monthly telephone calls + additional visits as needed; follow-up over 6 months | Not specified | 1+ face-to-face visits (average 1.86) + monthly telephone calls (average 2.48) | Diabetes self-management education; medication management (assessment of adherence, pillbox use, insulin injection technique); medication regimen appropriateness; drug-related problem identification and resolution; individualized diabetes education; medication counseling; adverse event surveys; cognitive assessment (MMSE); depression screening (TGDS-S); referral to other care team members |
| Ramadas 2018 [39] | Remote; web-based; individualised; self-paced | Web-based system with study nutritionist available via website for queries; developed using systematic six-step approach | Yes (Transtheoretical Model [TTM] and Stages of Change [SOC] construct; stage-personalised intervention; recommendations based on DSOC) | Personalised recommendations based on DSOC; 12 fortnightly lesson plans; log-in reminders via e-mail; text message reminders for non-login; e-mail queries to study nutritionist; process evaluation for intervention adherence and program reception | Fortnightly lesson updates over 6 months (12 lesson plans total) | Average time spent per login: approximately 12 minutes per lesson plan | 12 lesson plans over 6 months (fortnightly) | 12 dietary lesson plans covering: Sugar; Fruits and vegetables; Eating out habits; General recommendations from Malaysian CPG, European Association for the Study of Diabetes, American Diabetes Association, and Malaysian Medical Nutrition Therapy guidelines. Culturally adapted content: Local dishes with culturally and religiously-sensitive options |
| Mohammadi 2018 [40] | Face-to-face; likely group-based | Researchers/educators; educational booklet provided | Yes (Health Belief Model [HBM] with self-efficacy focus; constructs: perceived susceptibility, severity, benefits, barriers, self-efficacy) | Tailored self-efficacy education; educational booklet designed based on HBM; face-to-face interviews for data collection; phone calls allowed for questions during follow-up; diabetes quality of life questionnaire | 8 sessions over 3 months | 2 hours per session | 8 sessions | Diabetes and its complications; self-care and self-efficacy behavior; physical activity; healthy diet; medication adherence; self-monitoring of blood glucose level. HBM constructs: Perceived susceptibility; perceived severity; perceived benefits; perceived barriers; self-efficacy. Educational booklet covering diabetes definition, types, risk factors, symptoms, complications, main aspects of self-care, dietary management, importance of physical activity |
| Tourkmani 2018 [41] | Face-to-face; multidisciplinary; individual-based; primary care setting | Multidisciplinary team: senior family physician, clinical pharmacy specialist, dietician, diabetic educator, health educator, social worker | No | Weekly or bi-weekly team meetings to review eligibility and care plans; case manager responsible for arranging appointments and evaluating compliance/adverse effects; patient-based strategies: frequent clinic visits, frequent monitoring, multidisciplinary communication/coordination, additional diabetic education and dietetic advice, self-management promotion, home blood glucose monitoring booklet, insulin titration, medication adherence encouragement, social support, patient reminders, telephone calls; at least weekly appointments in first 3 months; patients seen at least once by all team members | At least weekly appointments in first 3 months; then as needed; median follow-up 10 months | Not specified | Multiple visits over median 10 months; visits to case manager, diabetes educator, health educator | Standard care per ADA guidelines but intensified with consideration for individual clinical and social factors; diabetic education; dietetic advice; self-management promotion; home blood glucose monitoring; insulin titration education; medication adherence counseling; social support; patient reminders; telephone call support. Eligibility criteria for program: poor glycemic control; failure to respond to insulin dose >2 units/kg or >200 units; inadequate insulin adherence; uncontrolled hypertension/hyperlipidemia with maximum medications; comorbidity; inadequate continuity of care |
| Jiang 2019 [42] | Face-to-face; group-based (4-8 patients per group); outpatient setting | Trained education nurses + physicians | Yes (Self-efficacy theory; four sources: performance accomplishments, vicarious experience, verbal persuasion, physiological/emotional arousal) | Group sessions (4-8 patients); goal setting progressively; positive feedback; practicing; experience sharing; peer modelling; demonstration and return-demonstration; verbal encouragement; consultations; peer support; evaluation and recording of biomedical indicators; homework; review and reinforcement; follow-up every 3 months (face-to-face/telephone); supportive written materials | 4 weekly sessions (60-90 min each) + follow-up every 3 months | 60-90 minutes per session | 4 weekly sessions + follow-up contacts every 3 months | Session 1: General knowledge on diabetes; self-monitoring; significance of managing diabetes; self-introduction and discussion; recording diet. Session 2: Diet; advantage of reasonable weight; categories of nutrients and calories (using food pictures); calculate daily diet; set goals; make diet plan. Session 3: Physical activities; regular physical activity; foot care and foot exercise; set goals; make exercise plan. Session 4: Regular examinations; sick-day tips; regular examinations; medication; encourage goal accomplishment; regular follow-ups. Cross-cutting: Diabetes knowledge; self-management behaviours; self-efficacy enhancement strategies incorporated throughout |
| Trento 2020 [43] | Face-to-face; group-based + individual consultations; multidisciplinary | Multidisciplinary team; physician (PF) for clinical management; group facilitators with pedagogic expertise | Yes (Self-management education model; adult education principles; patient-centered; peer education; methodological principles of adult education; focus on motivational aspects, emotional regulation, acceptance of diabetes, coping strategies, narration of real life situations) | Role-playing with real life simulations; patients express opinions and share life experiences; peer education; sharing and dialogue; group dynamics; individual consultations with physician after each group session; yearly screening for complications | Series of 7 sessions/modules held every 3-4 months, repeated twice over 4 years | 60 minutes per group session + individual consultations with physician after each session | 14 sessions over 4 years (7 modules × 2 cycles) | Self-management education; lifestyle attitudes; importance of pharmacological medication; patient adherence to anti-hypertensive and lipid-lowering prescriptions; healthy eating; physical activity; medication adherence; coping strategies; emotional regulation; acceptance of diabetes; narration of real life situations; problem identification and sharing; peer learning strategies |
| Guner 2020 [44] | Blended: face-to-face group-based education + SMS reminders (remote); primary care/FHC setting | Researcher/educator; delivered education at FHC education room | No | Power point presentations; face-to-face education; Education Booklet for Patients with Type 2 DM distributed; Diabetes Identity Card provided (from Diabetes Association); SMS reminders sent twice weekly for 6 months (48 total messages); messages based on education module issues and WHO diabetes education messages | Two education sessions (covering 5 modules) + SMS reminders twice weekly for 6 months | Not specified | 2 face-to-face group sessions (covering 5 modules) + 48 SMS reminders over 6 months | Module 1: General Information about Diabetes/Physiopathology of Diabetes. Module 2: Medical Nutritional Treatment. Module 3: Blood Sugar Regulatory Drugs other than Insulin in Diabetes. Module 4: Acute and Chronic Complications of Diabetes. Module 5: Physical Activity/Exercise in Diabetes and Life with Diabetes. SMS content: Blood glucose monitoring; blood pressure monitoring; medication adherence; physical activity (walking 30 min daily); foot care; dietary variety; fluid intake; weight/waist circumference monitoring; oral/dental health; flu vaccination; smoking cessation. Booklet: Education Booklet for Patients with Type 2 DM. Card: Diabetes Identity Card provided. |
| De la Fuente Coria 2020 [45] | Face-to-face; individualised; primary care setting; with family member/caregiver | One trained primary care nurse with >10 years of experience in type 2 diabetes education | Yes (Empowerment model; based on model of empowerment: promoting participation and strengthening person's autonomy; motivational interviewing techniques; goal setting) | Motivational interviewing techniques; goal setting; educational brochure containing contents and control objectives; self-management booklet; family member/caregiver attendance required; telephone calls for active recruitment of non-attenders; educational reinforcements after 12 and 18 months | 6 face-to-face sessions over 6 months (first phase) + reinforcement sessions at 12 and 18 months; follow-up at 24 months | 30 minutes per session | 6 initial sessions + 2 reinforcement sessions = 8 total sessions over 24 months | Based on American Diabetes Association/AADE7 Self-Care Behaviors: Basic knowledge of diabetes; healthy eating; physical activity; self-monitoring of blood glucose; medication; risk reduction; problem solving; effective coping. Additional: Educational brochure with control objectives; self-management booklet; goal setting |
| Tanaka 2021 [46] | Face-to-face; individual-based; physician-led; outpatient hospital setting | Physicians | No | JADEC Diabetes Education Card System; SMBG readings analyzer providing comprehensive summary statistics, listings and graphical plots of blood glucose profiles; JADEC self-management notebook for patients to record SMBG values; physician review of SMBG values with participants at each visit; physicians delivered necessary information to achieve optimal glycemic control | At baseline, 2, 4, and 6 months (4 visits over 6 months) | Not specified | 4 physician visits over 6 months | JADEC Diabetes Education Card System Program (71 guidance points): Essential knowledge to self-manage diabetes; target fasting and post-prandial glucose levels; lifestyle modifications; insulin dose adjustments based on SMBG patterns; SMBG analyzer feedback: graphical plots of daily glucose fluctuations; summary statistics; listings of blood glucose profiles; physicians provided feedback on SMBG values |
| Sanaeinasab 2021 [47] | Face-to-face; likely group-based (not explicitly stated as individual); outpatient clinic setting | Not explicitly specified (likely health educators/researchers delivering the SHEP model-based program) | Yes (Structured Health Education and Promotion [SHEP] model; a comprehensive systematic health education and promotion model) | Structured educational program; six educational sessions; lectures; group discussions; practical demonstrations; question-and-answer sessions; educational materials provided | Six sessions over 45 days | Not specified | Not specified | SHEP model-based comprehensive health education: Healthy lifestyle promotion; glycemic control; diabetes management; nutrition/dietary advice; physical activity/exercise; stress management; medication adherence; self-care behaviours; lifestyle modification strategies; health promotion behaviours |
| Pai 2021 [48] | Blended: face-to-face + remote; individual-based; outpatient shared care setting | Trained, experienced researcher; focus group input from diabetes department physicians, pharmacists, dieticians, diabetes educators, and physical-education teachers for program development | No | Self-learning digital videos; interactive diet-calculation software; wearable pedometer; monthly 10-minute phone consultations for tracking and reminders; focus group development; content validity assessed by experts (CVI>0.8); one-on-one health education (20-40 min) | One-on-one education (20-40 min) + self-learning videos at home + monthly 10-minute phone consultations for 6 months | 20-40 minutes (one-on-one education); 10 minutes (monthly phone consultations) | 1 one-on-one session + self-learning videos + 6 monthly phone consultations | Self-learning digital videos: Introduction to T2D; action mechanisms of medications; adverse effects; management of side-effects; self-monitoring of blood glucose level; instructions for physical activity and pedometer use. Interactive diet-calculation software: Daily calorie intake; nutritional evaluation; dietary suggestions; food pictures for consistency. Phone consultations: Tracking self-management behavior; addressing difficulties; reminders. Other: Lifestyle modifications; foot care; blood glucose self-monitoring |
| Omura 2022 [49] | Face-to-face; individual-based; dietitian-led; outpatient hospital setting | Dietitians | No | Dietary assessment using brief-type diet history questionnaire; computerized feedback reports summarizing individual food/nutrient intakes with individualized advice comments; standardized algorithms and advice schedule; for misreporters, additional simple checklist of major foods consumed over a week | Three 30-minute face-to-face sessions at 1, 3, and 5 months from study entry | 30 minutes per session | 3 sessions over 6 months | Individualized group: Individual nutritional profile derived from BDHQ; comparison with Japanese Dietary Reference Intakes; individual food group intakes profile; anthropometric profile and optimizing total energy intake; not skipping breakfast. Conventional group: Generalized pamphlets with pictures of hospital model menus; explanations for major food groups/nutrients using textbook |
| Ağralı 2022 [50] | Blended: face-to-face group-based education + telephone counseling; primary care setting | Researchers | Yes (Health Belief Model [HBM]: perceived susceptibility, severity, benefits, barriers, self-efficacy; integrated with health literacy-based approach) | HL-based approach; group education with ice-breaking activities, videos, photos, posters, presentations, activities; HL-tailored booklets; phone counseling for 6 weeks; expert panel for booklet development; education sessions framed with HBM sub-dimensions; stratified randomization by HL level; small group sizes (10-13 per group) | 6 weekly group sessions (45-60 min each) + 6 weekly phone counseling sessions + 12 weeks follow-up | 45-60 minutes per group session; phone counseling: not specified | 6 group sessions + 6 phone counseling sessions = 12 total contacts over 12 weeks; plus 12-week follow-up | Week 1 (Perceived Susceptibility): Definition of diabetes, risk factors, symptoms. Week 2 (Perceived Benefits/Self-efficacy): Diabetes management; diet; physical activity/exercise; medication/insulin treatment. Week 3 (Perceived Severity): Acute and chronic complications; effects of diabetes in the body. Week 4 (Self-efficacy): Health check-ups; self-glucose monitoring; blood pressure and lipid targets; oral/dental health; foot care; immunization; follow-up. Week 5 (Perceived Barriers): Special situations (religious practices, legal rights, travel, working life, skincare, sexual life, emotional/ social life, communication/ relationships in families). Week 6 (Self-efficacy): Diabetes care skills; blood sugar and blood pressure monitoring; foot care; oral/dental health care; skincare; insulin administration. Booklets: HL-tailored supportive detailed information on all topics. Phone counseling: Structured questions on medication regularity/timing, main meals/snacks, exercise, booklet reading |
| Feng 2023 [51] | Blended: remote (WeChat official account, messages, Moments) + face-to-face; family-based; community health center setting | Family practitioners and doctors in prevention/health section; research team developed intervention articles | Yes (Knowledge-Attitude-Practice [KAP] model + Health Belief Model [HBM]; conceptual framework: eHealth family intervention improves family members' knowledge and risk perception → helps patients with self-management → promotes community T2DM management participation → improves glucose control) | Official WeChat account ("Jiading Sugar Steward") with 3 modules; 38 intervention articles developed based on KAP + HBM; articles delivered via WeChat at different levels; in-person health education courses focused on skills practice; family member as designated supporter | Online: 38 articles delivered over 3 months + in-person health education every 3 months; total intervention duration 12 months | Online articles: not specified; In-person: as per National Standard for Basic Public Health Services | 38 online articles + 4 in-person sessions (every 3 months) + ongoing WeChat engagement | 38 articles across 4 domains: Diabetes knowledge; complications; risk; self-care activities (diet, exercise, medication, lifestyle, glucose testing, skills). KAP model topics: Basic knowledge → risk knowledge; behaviors for daily self-care; family members' attitudes on reminding patients. HBM topics: T2DM health complications (severity); benefits of self-care activities; risks of not performing activities. In-person: Risk perception-based health education courses focused on skills practice (e.g., proper exercise for glucose management; risks/benefits of self-care activities) |
| Renjbar 2024 [52] | Blended: face-to-face (group-based) + WhatsApp (virtual group support); individual notebooks; comprehensive health center setting | Medical doctor with PhD in Health Education and Health Promotion + MSc student in Health Education and Promotion; nutrition consultant and ophthalmologist arranged as needed | Yes (Behavioral Reasoning Theory [BRT]: behavioral intention, attitude, reasons for/against, values; designed to address limitations of Theory of Planned Behavior by including reasons for resistance/opposition to behavior) | WhatsApp group for intervention group; educational videos, clips, pamphlets, and tips provided after each session; notebooks for recording daily blood sugar levels; PowerPoint slides and images showing chronic complications; educational video clips with real examples; guest speaker sharing successful disease management experience; facilitated group discussions; brainstorming activities; Q&A sessions; emotional relief exercises; step-by-step guides; visual aids; interactive discussions | 8 weekly face-to-face training sessions over 2 months + WhatsApp group support between sessions | 45-60 minutes per session (Session 1: 60 min; Sessions 2-8: 45 min each) | 8 face-to-face sessions + ongoing WhatsApp group support | Session 1: Diabetes definition, types, role of insulin, symptoms, risk factors, complications, self-management behaviors, blood sugar monitoring methods, exercise, diet management (carbohydrate counting, plate method). Session 2: Benefits of self-management, preventing complications, attitude change, overcoming obstacles. Session 3: Social impact, subjective norms, social influence, family/doctor/ healthcare worker perspectives. Session 4: Perceived behavioral control, step-by-step self-management guide, practical methods. Session 5: Reasons for resistance, identifying solutions, practical health management suggestions. Session 6: Solutions to facilitate self-management, exercise facilitation, blood sugar measurement, guest speaker. Session 7: Consequences of actions, emotional relief, positive attitude maintenance. Session 8: Review of previous sessions, reasons for/against self-management, promoting adoption. Overall: Diabetes self-management education (DSME) including lifestyle changes, glucose management, diet, physical activities, medication adherence, foot care, blood sugar monitoring |
| Zainudin 2024 [53] | Face-to-face; individual-based; educator-led; hospital outpatient clinic setting | Diabetic educators; two educators per group; educators in SC group used standard flip chart and insulin pen models | No | USM-IAM module; SMBG diaries; insulin pen models; flip charts; handouts with insulin optimization instructions; educator phone numbers provided for questions; participants encouraged to ask questions and discuss practical solutions; module given to participants to keep and refer to freely | Counselling sessions at baseline, 3 months, and 6 months (3 sessions over 6 months) | Not specified | 3 counselling sessions over 6 months | USM-IAM module topics: Diabetes and insulin; Nonadherence to insulin; Insulin side effects; Problems with insulin injections; Negative attitude towards insulin; Expensive cost of monitoring; Wrong perception of insulin; Myths on insulin; Empowering diabetes self-care; Fasting safely with insulin therapy. Standard counselling (SC group): Blood sugar targets; insulin injection techniques; insulin dose modification; recognising and managing hypoglycaemia; SMBG diaries |
| Terkes 2024 [54] | Remote; web-based; self-paced; individualised; with email reminders | Researchers; website with online forum for patient questions; no direct educator involvement specified beyond programme development | No | Web-based education website with free unlimited access; unique login credentials for each participant; online forum for questions; email reminder messages twice weekly for 3 months; website notifications for emergencies; routine maintenance with web-based application throughout intervention period | 3-month web-based education programme + email reminders twice weekly | Not specified | Self-paced web-based education over 3 months + 24 email reminders (2 per week × 12 weeks) | Web-based education content: Not detailed in the abstract/published text; based on literature review; likely includes diabetes self-management, blood glucose monitoring, lifestyle modifications. Website features: Online forum for questions; emergency notifications; reminder messages. Email reminders: Supporting adherence to prescribed practices and guidelines, ultimately leading to lifestyle changes |
| Asmat 2024 [55] | Face-to-face; individual-based; nurse-led; outpatient department setting; home visit; culturally tailored for South Asian (Pakistani) population | Principal Investigator + Research Assistants; nurse-led intervention | Yes (Social Cognitive Theory; culturally tailored; based on American Association of Diabetes Educators self-care behaviors [AADE7]) | Eight weekly face-to-face individualized education, counseling, and behavioral training sessions; booster session; home visit; self-care guidebook in Urdu; motivational videos; real-time stories of successful patients as role models; six-step behavior modification process for self-efficacy enhancement; phone calls and SMS reminders during follow-up; comprehensive manual of operations; checklists for protocol compliance; Urdu translated/validated instruments (U-DMSES, U-SDSCA) | 8 weekly sessions + 1 booster session + 1 home visit over approximately 10 weeks; follow-up at 3 months post-intervention | 60-90 minutes per session | 8 weekly sessions + 1 booster session + 1 home visit = 10 contacts over ~10 weeks | Educational sessions: Disease fundamentals. Counseling sessions: Verbal persuasion; discussion on initiating and maintaining self-care behavioral change; motivational videos; real-time stories of successful patients as role models. Behavioral training: Six-step behavior modification process targeting four self-care behaviors. Booster session: Reflection on learning; feedback on self-care performance; behavioral goals review with personalized reinforcement; follow-up plan. Home visit: Personal, sociocultural, environmental facilitators/barriers; family and social support observation. Materials: Self-care guidebook in Urdu |
| Lau 2024 [56] | Remote: CGM telemonitoring + virtual/phone visits; individualised; community-based recruitment | Registered nurse certified diabetes educator; delivered virtual visits guided by standard template | No | Continuous Glucose Monitoring (CGM) with remote telemonitoring; CGM data-sharing via LibreView account; two structured one-on-one phone/virtual visits; individualized review of CGM data with recommendations for self-management; standard visit template; participants advised to double-check unusual/alarming glucose values against capillary blood glucose if available | 6 weeks of CGM use + 2 virtual educator visits (weeks 1-2 and 5-6); follow-up at 12 weeks | Not specified | 2 structured virtual diabetes educator visits | General information about type 2 diabetes and its treatment; individualized review of modifiable lifestyle factors affecting glycemic management; individualized review of CGM data with recommendations for self-management; (rarely) medication issues identified and forwarded to usual provider. Enhanced usual care: Identical content without CGM review. |
| Kim 2024 [57] | Blended: face-to-face + telephone (optional); individual-based; educator-led; hospital outpatient setting | Diabetes educators at each centre | No | Intermittently scanned CGM for 24 weeks; structured education programme on adjusting insulin dose and timing according to graphical patterns in CGM; individual education; digital patient education platform (iKooB); education at baseline, 4, 8, 12, and 18 weeks; blinded CGM for 2 weeks before baseline and for control group 2 at weeks 22-24 | Education at baseline, 4, 8, 12, and 18 weeks; CGM use for 24 weeks | Total structured education time: 3.12 ± 0.95 hours per participant (mean) | 5 education sessions over 24 weeks | Ideal basal insulin dose results in flat glucose trend overnight within target range; appropriate dose and timing of pre-meal rapid-acting insulin bolus results in postprandial glucose excursion ≤10.0 mmol/L with return to target over 4 hours; if postprandial glucose excursion above target but returns after 4 hours → adjust timing of insulin injection and/or meal composition; if postprandial glucose excursion does not return after 4 hours → adjust dose of insulin and/or meal composition. |
| Zhang 2024 [58] | Face-to-face; group-based (8-10 patients per group); nurse-led; primary care practice setting | Medical staff from each primary care practice; received one-day face-to-face training; passed assessment before commencing intervention | Yes (Nudge Theory; based on Thaler and Sunstein's nudge concept: predictable behavior change through choice architecture, environmental cues, and "gentle" rather than coercive means; designed to trigger intuitive rather than rational cognitive system) | Life-size food cards (100 kcal/portion except vegetables); "traffic light" classification cards (green/yellow/red); illustrative wall charts; diabetes diaries for self-monitoring; pre-class/post-class exercises; goal setting and public commitment; personalized feedback; visual "substitution rule" (3 steps); emotional arousal signs; reinforcement of key knowledge; reminders | Weekly sessions for 4 weeks (60-90 min each) + 3-month follow-up | 60-90 minutes per session | 4 weekly sessions | Balanced calorie-restricted diet per Chinese T2DM guidelines; life-size food cards (100 kcal/portion); "traffic light" classification (green=eat more, yellow=eat half usual amount, red=best not to eat); three golden dietary rules for weight loss; visualized 3-step "substitution rule" (pick out food cards for whole-day intake → replace higher-calorie cards with lower-calorie → classify using traffic light scheme); goal setting and public commitment; diabetes diary for self-monitoring |
| Hou 2024 [59] | Blended: face-to-face group-based education + text-messaging support (remote); community setting | Well-trained health education specialists; registered healthcare workers in communities | No | Culturally tailored DSME; text-messaging service every 2 weeks; colourful, well-illustrated educational handbook; logbooks for weekly lifestyle recording; participants encouraged to send messages to healthcare workers; expert assessment of curriculum content; content delivered at first week of every month over 6 months | 6 monthly sessions (120 min each) over 6 months + text messages every 2 weeks (12 messages) + logbook weekly recording | 120 minutes per session | 6 monthly sessions + 12 text messages + weekly logbook recording over 6 months | Six core sessions: Understanding diabetes; Diabetes complications; Diabetes therapy; Diabetes self-management; Physical activity, Eating, and Drinking; Psychological regulation. Text messages: Friendly delivery every 2 weeks; core messages on lifestyle behaviour, medication reminders, blood glucose monitoring prompts. Handbook: Colourful, well-illustrated educational handbook for home reference. Logbook: Weekly recording of physical activity, sedentary lifestyle, sleep, food intake, glucose-lowering medication adherence, blood glucose monitoring frequency |
| Pourmohammad 2024 [60] | Face-to-face; group-based (10-15 patients per group); health service center setting | Health educators/researchers | Yes (Theory of Planned Behavior [TPB]: attitude, subjective norms, perceived behavioral control, behavioral intention) | Direct interactive methods; indirect methods; verbal persuasion; teaching self-care behaviors in small, simple, applicable steps; key/influential people invited to final session to support patients; educational content tailored to pre-test results; special educational content according to patients' needs; health protocols for COVID-19 | 5 consecutive weekly sessions (45 minutes each) over 5 weeks | 45 minutes per session | 5 weekly sessions | Session 1: Generalities about diabetes (definition, types, signs/symptoms, complications). Session 2: Prevention and control of diabetes (non-pharmacological interventions); physical activity (importance, consequences, benefits) targeting attitudes. Session 3: Review of previous sessions; food groups and calories; specific diabetes diet; effect on blood sugar control; benefits of changing meal volume/number; targeting attitudes and behavioral intentions towards diet. Session 4: Verbal persuasion; self-care behaviors in small, simple, applicable steps to increase self-efficacy and perceived behavioral control; reducing barriers. Session 5: Review; importance of self-care behaviors (blood sugar measurement via glucometer, regular anti-diabetic drugs, continuous foot care, smoking avoidance, stress management); key/influential people invited to support patients. Overall: TPB constructs applied throughout |
| Zhao 2024 [61] | Face-to-face; likely individual-based or group-based (patient-centered); hospital outpatient setting | Not explicitly specified | Yes (Patient-centered health education model; based on patient-centered care principles where patients foster autonomy in managing their health; transition from doctor-centered to patient-centered approach) | Patient-centered approach; health education activities empowering patients to foster autonomy in health management; focus on knowledge, attitude, practice, and self-efficacy; dietary plans, physical activity, medication adherence education | Not specified | Not specified | Not specified | Patient-centered health education covering: Importance of maintaining a healthy lifestyle; dietary plans; physical activity; medication adherence; disease knowledge; attitudes towards disease; self-efficacy; blood glucose management; lipid management. Specifically for type 2 diabetes with hyperlipidemia: Glucose and lipid metabolism management; reducing cardiovascular risk; promoting self-care; enhancing capacity to control factors shaping health-related decisions |
| Ye 2024 [62] | Blended: remote (WeChat group, daily posts, Q&A) + face-to-face (offline follow-up every 3 months); multidisciplinary team; group-based + individualised | Multidisciplinary team: 2 endocrinologists, 1 cardiologist, 3 nurses | No | WeChat group; daily posts on basic knowledge about diabetes and hypertension; daily management norms; Q&A for patient confusion/misunderstandings; guidance on coping with hypoglycemia; lifestyle guidance; encouragement for patients to share personal experiences within the group; regular offline follow-up visits every 3 months | Daily WeChat posts and interaction + offline follow-up every 3 months over 26 weeks | Not specified | Daily WeChat communication for 26 weeks + 2-3 offline follow-up visits | Basic knowledge about diabetes and hypertension; daily management norms for diabetes and hypertension; dietary guidance; exercise guidance; medication adherence; blood glucose monitoring; blood pressure monitoring; coping with hypoglycemia; correcting unhealthy behaviors; improving disease knowledge; lifestyle modification; self-management skills; patient experience sharing |
| Mateos 2025 [63] | Remote; digital; multi-platform (WhatsApp preferred; also email, Skype, telephone, video conferencing, Instagram webinars); individualised; with Digital Diabetes Coach support | Digital Diabetes Coach + multidisciplinary team | No | Digital platforms selected based on patient preference; real-time or recorded webinars through Instagram; weekly comprehensive diabetes education sessions (30-45 min); 2-way communication system for on-demand questions; pedometer activation on mobile phones for step count data sharing; patients encouraged to share weight, capillary blood glucose, and step count data; educational materials from scientific societies and endorsed in diabetes education; Digital Diabetes Coach providing unidirectional and bidirectional interaction | Weekly education sessions (30-45 min) + on-demand digital support for 6 months | 30-45 minutes per weekly session | Weekly sessions over 6 months (~24 sessions) + ongoing on-demand digital support | Weekly sessions: Proper injection techniques; dosage adjustments for medications; strategies for managing adverse events; personalized physical activity recommendations; blood glucose profiling and interpretation; handling hypoglycemia/hyperglycemia; portion-controlled dietary plans; macrovascular and microvascular complications. Digital support: Healthy lifestyle habits; dietary support; optimization of physical activity; insulin titration; therapeutic targets; self-care. Materials: Validated material from scientific societies, external companies endorsed by these societies, and centre-developed materials |
| Bretschneider 2025 [64] | Remote; mobile app (iOS/Android); self-paced; individualised; with peer support group and personal consultant for technical support | Mobile health app (Vitadio) + personal consultant; digital health app (DiGA) certified as Class I medical device | No | Educational modules; daily tasks with automated messages; personalized SMART weekly goals; peer support group feature; photo-based nutrition diary with automatic feedback; step tracking via Google Fit/Apple Health synchronization; weight logging; blood glucose logging; automated motivational messages; personal consultant for technical support | Daily app engagement over 6 months (intensive phase: 3 months; sustain phase: 3 months); weekly goals; average use: 95 days of 180 (every other day) | Self-paced (app-based; daily tasks) | Self-paced over 6 months; average number of days used: 95 (SD 63); 81% completed all core education materials; average 189 meal photos per participant | Educational modules: Weight management; balanced nutrition; glycemic control; physical activity; sleep; stress management. Daily tasks: Automated messages providing structured pathway through program. SMART weekly goals: Dietary habits; activity levels; self-care practices. Monitoring: Photo-based nutrition diary; step tracking; weight logging; blood glucose logging. Peer support: Community feature for motivation and adherence. |
| Mihevc 2025 [65] | Remote; mHealth home telemonitoring; Bluetooth-connected devices; cloud platform; primary care setting | General Practitioner + nurses | No | Home telemonitoring package; Bluetooth data transmission to mobile app; 4G/5G data transfer to telemedicine cloud platform; colour-coded alert system; data encryption and GDPR compliance; 1-hour training by nurse at enrollment | BP: twice weekly; BG: once monthly over 12 months; abnormal results triggered more intensive monitoring | Not applicable (self-measurement at home; 1-hour initial training) | 1-hour initial training + ongoing telemonitoring for 12 months | Initial training: Measurement protocol for BP and BG; how to use telemonitoring devices; how to transmit data via Bluetooth. Monitoring: BP measurement protocol; BG measurement protocol. Alert system: Green = normal; Yellow = abnormal → 7-day BP profile or 1-day BG profile; Red = critically abnormal → immediate teleconsultation with GP. Treatment goals: Home BP <135/85 mmHg; fasting BG <7 mmol/L; postprandial BG <10 mmol/L |
| Zhang 2025 [66] | Blended: remote (telemedicine smart device, automatic data transmission, alerts, chat) + face-to-face (group education sessions every 2 weeks); multidisciplinary team; community-based | Multidisciplinary team: 8 doctors, 2 pharmacists, 20 nurses, 1 information engineer | Yes (Health Belief Model [HBM]: perceived susceptibility, perceived severity, perceived benefits, perceived barriers, cues to action, self-efficacy) | Xiaodu Smart Screen X8 telemedicine device; automatic health data transmission to centralised website; automatic alerts for abnormal blood glucose; nurse reminders if no data uploaded for >1 week; voice-recorded medication intake; personalised diet and exercise regimens generated by smart device; one-click medical alert; bidirectional chat function; face-to-face group education sessions every 2 weeks for 3 months; diabetes self-management handbook based on HBM; multimedia materials | Blood glucose self-measurement: ≥2×/week for 6 months; Group education: every 2 weeks for 3 months (6 sessions); Health care team reviewed data every 2 weeks; Pharmacists sent monthly medication management plans | 40-50 minutes per group education session | 6 group education sessions over 3 months + ongoing telemonitoring for 6 months | HBM-based group education sessions: Definition, symptoms, risk factors of T2D; benefits of using smart devices for diabetes management; impact of T2D on quality of life and physical health; diabetes self-management through smart devices; discussion of patient experience in self-management and device use, solutions to reduce barriers; mobilising family support, role models, increasing self-efficacy. Telemedicine: Smart device training; blood glucose monitoring (≥2×/week); medication management; diet/exercise guidance; automatic alerts; bidirectional communication; personalised diet and exercise regimens. Handbook: Diabetes self-management handbook based on HBM |
| Epriliawati 2025 [67] | Face-to-face; individual-based; hospital outpatient setting; non-medical professional-led coaching | Health coaches + nationally trained/certified diabetes educators | No | CARE coaching model (4 basic elements); individual coaching sessions (60 min every 2 weeks); 12 coaching themes; patient-centred approach; 12 standardised education sessions (60 min every 2 weeks) provided to both groups | Education: 12 sessions every 2 weeks (60 min each); Coaching: 12 individual sessions every 2 weeks (60 min each); Follow-up at 3 and 6 months | 60 minutes per education session; 60 minutes per coaching session | 12 education sessions (both groups) + 12 coaching sessions (intervention group only) = 24 sessions total over 6 months | Standard education (both groups): Introduction to DM; Diet and nutrition for DM patients; Physical activity and exercises; Medication treatment for diabetes; Self-monitoring blood glucose (SMBG); Hypoglycemia; Diabetic ketoacidosis; Chronic complications of DM; Diabetic foot; DM during fasting; DM during traveling; DM during illness. Health coaching (intervention group only): Life balance discovery; Holistic alignment; Healthy life vision; Mind balance; Emotional balance; Healthy state enhancement. CARE coaching model: Clarity; Awakening; Resolution; Empowerment |
| Li 2025 [68] | Blended: face-to-face group lectures + telephone follow-up + home visits + group mutual support; community-based; multidisciplinary | Principal investigator + community chronic disease management nurses (trained); physicians with ≥7 years clinical experience | Yes (Behaviour Change Wheel [BCW] theory + COM-B model: Capability, Opportunity, Motivation_Behaviour; combined with Teach-Back method) | Health education lectures (6 sessions, 60-90 min each, monthly); Teach-Back method); telephone follow-up (15-20 min, monthly, using teach-back method); face-to-face communication; home visits (quarterly, 2 sessions); group mutual support (2-3 nearby patients); rewards; blood pressure/blood glucose monitoring record cards; health education manuals; PowerPoint presentations; short videos on complications; demonstration and correction of measurement techniques; experience-sharing sessions with successful patients; national public health policy guidance; Community Health Service Center consultations | Monthly health education lectures for 6 months + monthly telephone follow-ups + quarterly home visits + ongoing group mutual support | 60-90 minutes (health education lectures); 15-20 minutes (telephone follow-ups); 15-20 minutes (home visit interviews) | 6 monthly lectures + 6 monthly telephone follow-ups + 2 quarterly home visits + ongoing group support | Lecture 1: Causes and treatment of HTN and T2DM (diet, exercise, self-monitoring, medications, complications, emotions, emergency management). Lecture 2: Self-management success stories; factors affecting blood pressure/glucose monitoring; correct measurement/recording; demonstration/correction of techniques. Lecture 3: Dietary modification (food combinations, mealtime choices, common misconceptions). Lecture 4: Exercise precautions; simple/safe exercises (walking); experience sharing. Lecture 5: Medication instructions/adverse effects; simplest effective medication; emotional impact on HTN/T2DM; emotion relief techniques. Lecture 6: Complications impact (short videos); prevention/recognition/early management of acute/chronic complications. COM-B/BCW-based: Education, persuasion, training, enablement, restriction, incentivisation, modelling, environmental restructuring. Teach-Back: Iterative comprehension assessment and reinforcement |
| Terkes 2025 [69] | Remote; distance education via Zoom; synchronous (live) with recordings for absent participants; individualised question support via WhatsApp | Researchers; delivered structured distance education via Zoom | No | WhatsApp group for education schedule information and participant questions; Zoom sessions (weekly, 40 min each, for 4 weeks); interactive presentations with question-answer periods; recorded sessions sent to participants who could not attend; follow-up at 3 months; participants could send questions via WhatsApp at any time | 4 weekly Zoom sessions (40 min each) over 4 weeks | 40 minutes per session | 4 weekly Zoom sessions | Four weekly sessions covering: Disease knowledge; Nutritional therapy; Physical activity/exercise; Insulin use; Blood glucose monitoring; Diabetes complications; Foot care; Lifestyle adjustments. Content developed based on: National and international diabetes management guidelines; Expert panel review; Pilot patient feedback for content comprehensibility, engagement, and practical usability |
| Sjoblom 2025 [70] | Remote; smartphone app; self-paced; self-delivered; Swedish language | Smartphone application; no active educator involvement; app developed specifically for the study; participants could contact study personnel for technical issues | Yes (Health Belief Model + Stages of Change Model + Social Cognitive Theory; behavior change techniques: goal-setting, educational information, self-monitoring, feedback) | Weekly topics with educational information; weekly tasks related to topic; healthy recipes; practical advice; short fun facts; reminders; weekly evaluation; automatic prompts/notifications when new activities available; self-paced personal progress; app downloaded with study personnel assistance | 12-week program; weekly topics; participants encouraged to use app daily | Self-paced (app-based; daily use encouraged) | 12 weekly topics over 12 weeks (136 total app activities); average completion: 71.3% of activities | 12 weekly topics (Swedish national dietary guidelines): Week 1: Healthy food patterns; Weeks 1,2,5: Fruit and vegetables/root vegetables/berries; Week 3: Total energy; Week 4: Sucrose; Week 6: Carbohydrates; Weeks 6,7: Fiber and whole grains; Week 8: Legumes; Week 9: Red/processed meat and saturated fat; Week 10: Unsaturated fat and fish/seafood; Week 11: Sodium/salt; Week 12: Sugar-sweetened beverages and fruit juice. Features: Educational information; weekly tasks; healthy recipes; practical advice; short fun facts; reminders; weekly evaluation |
| Li 2025 [71] | Face-to-face; individual-based; GP-led; community health centre setting; with remote client for progress recording | General Practitioners (GPs) with similar medical capacity, professional title, clinical experience (>3 years), weekly work hours (>32h); all received comprehensive training before study; research assistants reviewed recordings/assessment forms to ensure 100% fidelity | Yes (CAPDCA model based on PDCA cycle management + KAP + Motivational Interviewing + Behavioural Change Theory + Teach Back Method; integrates learning competency assessment, MI, and behavioural change theory) | CAPDCA model (6 phases); teach-back method; motivational interviewing; learning competency assessment; collaborative physician-patient decision-making; modular teaching strategy with incremental goals; patient progress recording and uploading to remote client for physician adjustment; family/peer support; regular review of audio/video recordings; educational prescriptions; problem assessment forms; phased follow-up schedule (10 times over 18 months) | 10 follow-ups over 18 months (Stage 1: monthly; Stage 2: 2-monthly; Stage 3: 3-monthly); ongoing education | <15 minutes per consultation (per GP feedback) | 10 follow-up contacts over 18 months + ongoing CAPDCA cycles | CAPDCA model phases: Collection; Assessment; Plan; Do; Check; Aggrandisement. Overall: National Basic Public Health Service Standard + National handbook for prevention and control of diabetes in primary care |
| Isleyen 2025 [72] | Blended: face-to-face group education (hospital training room) + WhatsApp video call MI (remote, individual); nurse-led | Nurse researcher | Yes (Information-Motivation-Behavioural Skills [IMB] Model; integrated with Motivational Interviewing techniques) | Diabetes education booklet; group education (5-6 people per group, 4 sessions); PowerPoint presentations; insulin tools, hybrid arm, glucometer; teach-back methods; MI via WhatsApp video call (5 sessions, every 2 weeks, 30-45 min each); OARS techniques; goal setting; change plan development; appreciation and collaboration; summary of whole process | Education: 4 sessions (weekly); MI: 5 sessions (every 2 weeks); total intervention: 12 weeks | Education: ~60 min per session; MI: 30-45 min per session | 4 group education sessions + 5 individual MI sessions = 9 sessions over 12 weeks | Session 1: Definition of diabetes; incidence; types; treatment; diabetes management; healthy nutrition. Session 2: Waist/hip measurement; BMI calculation; physical activity/exercise. Session 3: Insulin use; self-monitoring of blood glucose; insulin administration demonstration. Session 4: Short/long-term complications and measures; daily life recommendations (skin care, oral/dental health, vaccination, sleep, smoking/alcohol, travel). MI sessions (WhatsApp video call): Session 1: Agenda setting, topic bubbles, OARS techniques, goal setting, change plan development; Session 2: Physical activity/exercise management, appreciation, collaboration; Session 3: Regular/correct insulin use; Session 4: Regular blood glucose measurement; acute/chronic complications; Session 5: Life with diabetes, summary of whole process, appreciation, feedback. Booklet: IMB model-based Diabetes Education booklet |
| Lo 2025 [73] | Face-to-face; group-based (3-4 participants per group); facilitated by certified diabetes educator; hospital setting | Certified diabetes educator | Yes (Health Belief Model [HBM]: perceived susceptibility, severity, benefits, barriers, cues to action; interactive learning through dialogue and peer discussion) | Conversation map; discussion cards containing myths and facts; facilitator guide with open-ended questions; small group sessions (3-4 PWD per group); peer interaction and dialogue; story sharing; role modelling; collaborative learning; self-reflection; facilitated group discussions; participants encouraged to suggest solutions; dispelling myths; open-ended discussion prompts aligned with HBM | Single 1-hour session | 60 minutes | 1 session | Living with Diabetes conversation map topics: Living with diabetes; diabetes self-management; diet and exercise for glycemic management; myths and facts about diabetes; complications; medication; blood glucose monitoring; lifestyle modifications; psychosocial aspects; goal setting. HBM-based discussion prompts: Perceived susceptibility; perceived severity; perceived benefits; perceived barriers; cues to action. Peer interaction: Experience sharing, story telling, role modelling, mutual learning, social support |
