# Supplementary material for: Effect of educational program for patients with type 2 diabetes: a systematic review and meta-analysis of randomized controlled trials
Source: Front Med (Lausanne). 2026 Aug 13;13:1860805. doi: 10.3389/fmed.2026.1860805 (PMC13518541; doi:10.3389/fmed.2026.1860805)
Supplement: Supplementary file 3 [file Table_3.docx]

Figure S1. Risk of bias in individual trial

Figure S2. The summary risk of bias for included trials
