# Supplementary material for: Effect of educational program for patients with type 2 diabetes: a systematic review and meta-analysis of randomized controlled trials
Source: Front Med (Lausanne). 2026 Aug 13;13:1860805. doi: 10.3389/fmed.2026.1860805 (PMC13518541; doi:10.3389/fmed.2026.1860805)
Supplement: Supplementary file 4 [file Table_4.docx]

Figure S1. Sensitivity analysis for HbA1c

Figure S2. Sensitivity analysis for FBG

Figure S3. Sensitivity analysis for 2hPG

Figure S4. Sensitivity analysis for BMI

Figure S5. Sensitivity analysis for SBP

Figure S6. Sensitivity analysis for DBP

Figure S7. Sensitivity analysis for LDL

Figure S8. Sensitivity analysis for HDL

Figure S9. Sensitivity analysis for TC

Figure S10. Sensitivity analysis for TG
