# Supplementary material for: Effect of educational program for patients with type 2 diabetes: a systematic review and meta-analysis of randomized controlled trials
Source: Front Med (Lausanne). 2026 Aug 13;13:1860805. doi: 10.3389/fmed.2026.1860805 (PMC13518541; doi:10.3389/fmed.2026.1860805)
Supplement: Supplementary file 5 [file Table_5.docx]

Figure S1. Meta-regression for HbA1c according to country (P=0.152)

Figure S2. Meta-regression for HbA1c according to sample size (P=0.179)

Figure S3. Meta-regression for HbA1c according to mean age (P=0.097)

Figure S4. Meta-regression for HbA1c according to male proportion (P=0.001)

Figure S5. Meta-regression for HbA1c according to baseline HbA1c (P=0.119)

Figure S6. Meta-regression for HbA1c according to follow-up duration (P=0.975)

Figure S7. Meta-regression for HbA1c according to delivery format (P=0.567)

Figure S8. Meta-regression for HbA1c according to session type (P=0.526)

Figure S9. Meta-regression for HbA1c according to theory-based intervention (P=0.593)

Figure S10. Meta-regression for HbA1c according to multidisciplinary involvement (P=0.035)
