# Supplementary material for: Effect of educational program for patients with type 2 diabetes: a systematic review and meta-analysis of randomized controlled trials
Source: Front Med (Lausanne). 2026 Aug 13;13:1860805. doi: 10.3389/fmed.2026.1860805 (PMC13518541; doi:10.3389/fmed.2026.1860805)
Supplement: Supplementary file 6 [file Table_6.docx]

Figure S1. Funnel plot for HbA1c

Figure S2. Trim and fill for HbA1c

Figure S3. Funnel plot for FBG

Figure S4. Trim and fill for FBG

Figure S5. Funnel plot for 2hPG

Figure S6. Funnel plot for BMI

Figure S7. Trim and fill for BMI

Figure S8. Funnel plot for SBP

Figure S9. Funnel plot for DBP

Figure S10. Funnel plot for LDL

Figure S11. Funnel plot for HDL

Figure S12. Funnel plot for TC

Figure S13. Funnel plot for TG

Figure S14. Trim and fill for TG
